# Comparative Environmental Assessment of Three Urine Recycling Scenarios: Influence of Treatment Configurations and Life Cycle Modeling Approaches

**DOI:** 10.1021/acs.est.5c09248

**Published:** 2025-09-24

**Authors:** Abdulhamid Aliahmad, Prithvi Simha, Björn Vinnerås, Jennifer McConville

**Affiliations:** Department of Energy and Technology, 8095 Swedish University of Agricultural Sciences, Box 7032, S-75007 Uppsala, Sweden

**Keywords:** life cycle assessment, eco technology, urine recycling, resource recovery, source separation

## Abstract

Urine recycling is an emerging promising approach for enhancing resource recovery and mitigating environmental impacts in sanitation systems. This study presents a comparative life cycle assessment (LCA) of a urine dehydration system implemented at three levels of decentralization: (i) toilet-level units within bathrooms; (ii) basement-level units serving multiple households; and (iii) centralized neighborhood-scale facilities using dedicated sewers for off-site processing. Each configuration is assessed using both consequential and attributional system models across five impact categories: global warming potential, acidification, freshwater and marine eutrophication, and cumulative energy demand. The basement-level system consistently shows the lowest impacts, with up to 50% lower global warming potential than the other configurations. Centralized treatment is the most energy-efficient per liter of urine treated, but the sewer infrastructure burden offsets this advantage. Sensitivity analysis shows that substituting sulfuric acid for citric acid and achieving >52% heat recovery can yield net-negative emissions at the basement level. The choice of the LCA system model strongly affects results: attributional with substitution yields net-negative impacts, whereas consequential provides more conservative but robust estimates. The findings underscore the need for methodological transparency in LCA and provide guidance for scaling sustainable decentralized urine recycling.

## Graphical abstract

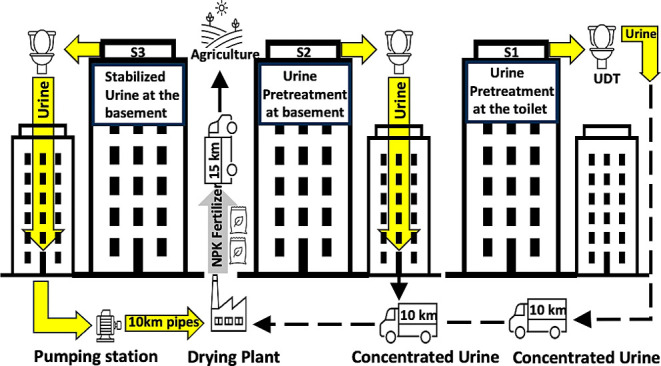

## 1 Introduction

Urine recycling is increasingly recognized as a strategy for supporting the transition toward more circular and sustainable sanitation systems.^1^ Conventional sanitation systems focus on end-of-the-pipe solutions, prioritizing pollution control over resource recovery and upstream solutions.^2^ Although some modern wastewater treatment plants (WWTPs) have begun to integrate resource recovery (e.g., phosphorus and energy), they are still limited and overlook valuable nutrients like nitrogen and potassium.^3^ Their effluents frequently contain some of these nutrients, which can contribute to ecological issues, such as eutrophication, when discharged into nearby aquatic ecosystems.^4^ Urine stands out because it makes up only a small portion of domestic wastewater, yet it contains most of the nutrients found in wastewater.^5^ Hence, source-separated urine presents a unique opportunity for nutrient recovery, specifically producing urine-based fertilizers that can serve as a substitute for synthetic fertilizers, thereby mitigating the environmental burden associated with both fertilizer production and conventional wastewater treatment. Additionally, this approach promotes a circular economy in nutrient management, enhancing sustainability in agricultural practices.^6,7^

In recent years, several innovative technologies for urine recycling have emerged.^8^ These technologies enhance urine recycling practices beyond traditional urine storage methods, which encountered many logistical challenges, such as difficulties in transporting high volumes of urine and storing it at collection sites and farms.^9^ The new urine recycling technologies apply alternative and advanced treatment processes that can effectively reduce volume while generating fertilizers with a higher nutrient content and reduced levels of contaminants. For instance, nitrification-distillation technologies yield concentrated urine-based liquid fertilizers,^10^ whereas dehydration technologies produce solid urine-based fertilizers.^11^ Solid urine-based fertilizers are particularly well suited for pelletization and can be readily integrated into agricultural systems that rely on existing machinery and large-scale farming practices. Consequently, they offer a highly viable solution for industrialized farming, allowing farmers to retain their current machinery and habits. Simha^12^ asserts that a solid urine fertilizer requires only 900 kg per hectare, compared to 15,000 kg of unconcentrated urine, assuming cereal crops need 90 kg N ha^–1^ and dried urine contains 10% N.

Several life cycle assessments (LCAs) have evaluated the environmental performance of urine recycling systems in comparison to conventional wastewater treatment systems. The environmental benefits of the direct application of stored urine have been assessed and shown in multiple studies.^13–16^ Decentralized urine diversion systems at the university scale have demonstrated environmental advantages in phosphorus recovery through struvite and potential pharmaceutical removal.^17,18^ Building-scale and centralized pretreatment using struvite precipitation and microbial electrolysis cells (MEC) showed significant reductions in environmental impacts, along with high phosphorus and ammonia recovery efficiency.^19^ The city-scale modeling of centralized urine treatment using struvite precipitation and ion exchange also indicated substantial reductions in greenhouse gas emissions, eutrophication, and water use.^20^ Centralized blackwater and urine systems incorporating struvite precipitation and transmembrane chemisorption (TMCS) outperformed conventional treatment in multiple environmental impact categories.^21^ Most recently, hybrid systems combining decentralized urine dehydration with blackwater management have been shown to outperform centralized treatment plants and other source separation systems due to their enhanced nutrient recovery and potential for fertilizer substitution.^22^ Collectively, this literature demonstrates the potential of urine recycling to mitigate the environmental burdens associated with conventional WWTPs, particularly through avoided nutrient removal processes, reduced methane and nitrous oxide emissions, and synthetic fertilizer substitution.

Despite these advances, two key gaps remain. First, little is known about how different urine treatment configurations and treatment locations, whether at the toilet, in the basement of a multistory building, or in a centralized neighborhood-scale facility, affect the environmental performance. Treatment location influences collection logistics, energy demand, emissions, and scalability, yet these context-specific trade-offs have not been systematically compared to guide decision-making and support technology scale-up. For instance, toilet-level treatment reduces the need for piping and is suitable for retrofitting older buildings^12^ but may require more energy and frequent maintenance.^23,24^ Basement-level treatment can process larger volumes and is generally more energy-efficient.^22^ Centralized treatment may offer the highest energy efficiency per unit of urine treated; however, it involves transporting urine through the sewer infrastructure, which introduces complexity and burdens that are often underrepresented in earlier LCAs.^25,26^ Second, few studies have critically examined how methodological choices in LCA—particularly the use of attributional versus consequential approaches—affect the interpretation of results for emerging sanitation technologies. These approaches are designed to answer different types of questions,^27^ and the choice between them significantly influences which inputs and system boundaries are included in the analysis.^28,29^ Aligning the LCA model with the study’s objectives is, therefore, essential for producing credible, transparent, and policy-relevant results. Inconsistencies in methodological choices across studies hinder meaningful comparison and limit the usefulness of LCA for guiding decision-making.

This study addresses both gaps by applying LCA to compare urine dehydration systems implemented at three treatment locations (toilet, basement, and centralized facility). It further contrasts attributional cutoff and consequential system models to evaluate how methodological choices influence results and their interpretation for decision-making. Specifically, the study asks: (1) how does treatment location impact the environmental performance of urine recycling systems? (2) which configuration, if any, achieves net-negative impacts across all assessed impact categories? and (3) how do attributional cutoff versus consequential models alter the interpretation of results and conclusions drawn for decision-makers? By integrating technological and methodological perspectives, this study provides actionable insights for the sanitation system design, LCA practice, and a broader transition toward sustainable nutrient management.

## 2 Materials and Methods

### 2.1 Study Scenarios

This LCA aims to evaluate the environmental performance of a urine recycling system under different treatment locations and modeling approaches. The case study focuses on five newly constructed residential buildings in a Swedish city, each comprising 10 apartments with an average of 2.5 capita per apartment, resulting in a total of 50 apartments and 125 capita. Three distinct urine recycling scenarios are analyzed based on the treatment location: the toilet, the basement, and a centralized treatment station. Each scenario is examined using two modeling approaches, consequential and attributional cutoff models, which are discussed in Section 2.2. The three urine recycling scenarios share several unit processes but exhibit distinct differences, particularly in urine collection, concentration, and transportation to the final drying facility. Figure 1 illustrates the unit processes involved in the three urine recycling scenarios.

**Figure 1:**
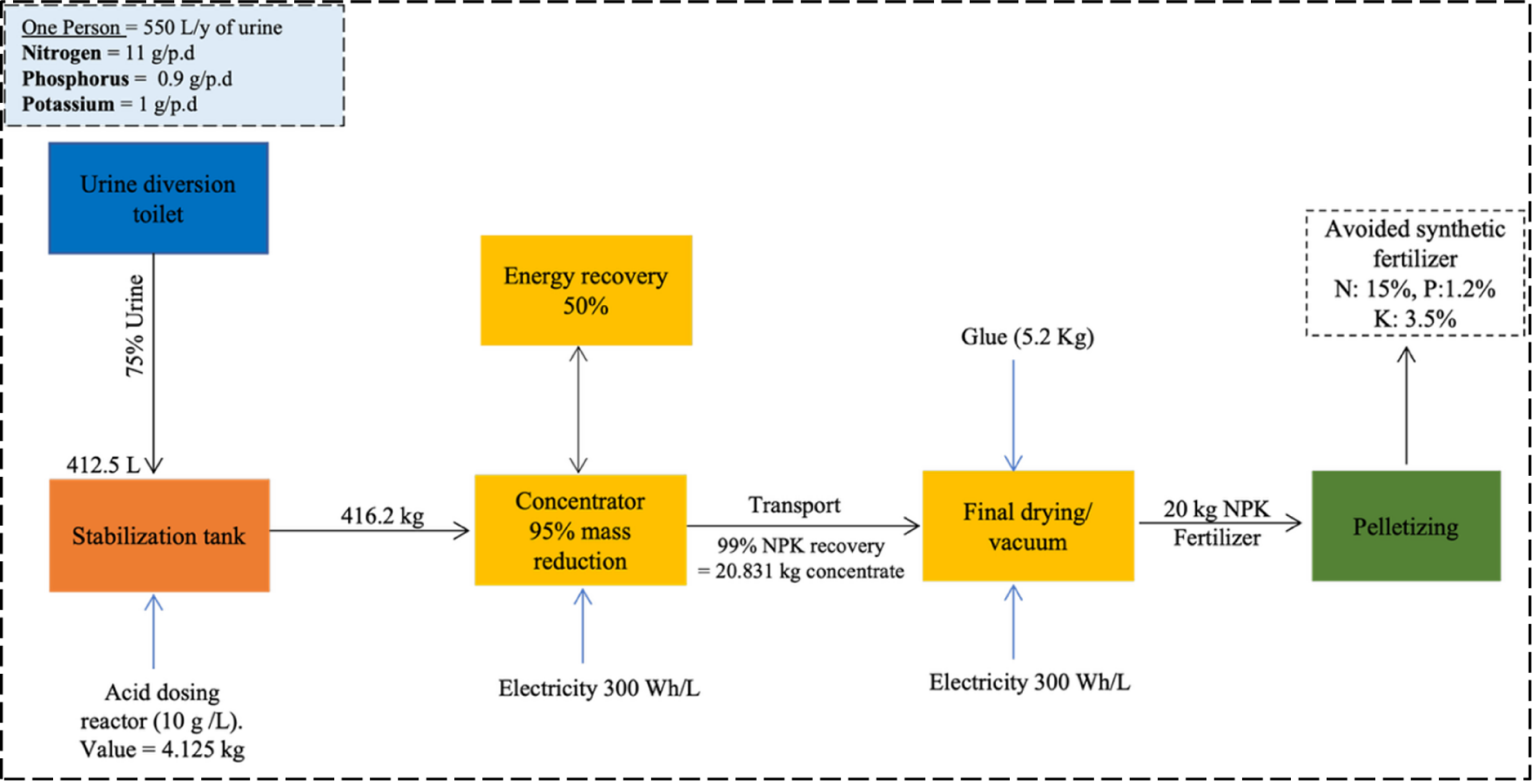
Schematic diagram of the primary unit process of the urine recycling system scenario (1) Energy recovery is achieved through heat recovery using a heat exchanger, which differs between the three scenarios. Each unit process is represented by a distinct color, which is used consistently throughout the study to facilitate comparison, particularly in the results.

Initially, urine is separately collected using a urine diversion toilet and subsequently stabilized by adding 10 g of citric acid per liter of urine to prevent enzymatic urea hydrolysis.^30^ The stabilized urine undergoes a concentration process that aims at reducing its volume through dehydration. This process varies slightly based on the scale and location of the treatment system. In a toilet-level configuration, the concentration is achieved via convective evaporation, where warm air (∼50 °C) is circulated over the stabilized urine using a fan and pump system. This method is compact and well suited for installation in bathrooms, as it does not require pressurized or complex equipment. It effectively removes over 90% of the water and has been validated in previous field studies (e.g., Simha^12^). In basement and centralized configurations, the bulk of the water is removed through distillation during the concentration step. This approach proves to be more energy-efficient for larger volumes and allows for the direct integration of heat exchangers for energy recovery. Once the urine is sufficiently concentrated, it is transferred to vacuum evaporation for final drying. This step is conducted under reduced pressure to lower the boiling point and preserve the nitrogen content. At this stage, organic binders are also introduced to facilitate pellet formation and to enhance product handling. Consequently, a second distillation step is not viable as the presence of these added materials alters the physical characteristics of the concentrate, making low-pressure drying a more suitable option. The dehydrated urine product generated in all three scenarios is a stable solid fertilizer containing approximately 15% N, 1.2% P, and 3.5% K (Figure 1), with ∼99% nutrient recovery from the collected urine. The stabilization process prevents urea hydrolysis, ensuring that no significant nutrient losses occur during the concentration, storage, or drying. Similar urine-derived fertilizers produced via this method have been successfully field-tested in Sweden and other countries, showing agronomic performances comparable to conventional mineral fertilizers when applied on an NPK-equivalent basis.^12^ Therefore, this LCA models the urine-based fertilizer as a complete substitute for synthetic fertilizers on a nutrient-equivalent basis. Readers are encouraged to review our previous LCA study for a more comprehensive understanding of the different unit processes and mechanisms involved.^22^

The first scenario, decentralized household treatment (S1—toilet-level), is illustrated in Figure S2 in the Supporting Information. In this scenario, urine is collected directly from the toilet, where it is generated, with the concentration unit installed within the same bathroom. This design allows for a direct connection from the urine-diverting toilet to the treatment unit via a short pipe. Urine is stabilized and concentrated daily, and the concentrate is stored within the unit for two months before being transported to the final drying facility. The unit is designed to accommodate urine output from a single apartment, factoring in routine inflow and allowing for a buffer volume to prevent overflow during periods of high use or unexpected inflow. Each capita produces 1.13 L of urine per day or about 550 L/year. With a capture rate of 75%,^31^ this results in 413 L collected per capita per year. The concentration process achieves a 95% mass reduction, yielding about 21 kg of concentrate per capita annually. Transport occurs six times per year (once every two months), with each trip covering a 20 km round trip to the drying facility, totaling 411 kg km per capita per year; see Table S12 in the Supporting Information. Once dried, 20 kg of the urine-derived fertilizer is delivered to a local farm to substitute for synthetic fertilizers. The energy requirement for the urine concentration process is 600 W-hours per liter (Wh/L). Each urine recycling scenario in this LCA incorporates heat recovery, which recovers a portion of the thermal energy and reuses it within the system. In the toilet scenario, to reduce electricity demand, heat recovery ventilation (HRV) is assumed, which is consistent with Swedish residential systems. These systems recover thermal energy from exhaust air and typically use it for space heating. Here, a portion of that recovered heat is assumed to prewarm the air entering the urine concentration unit (to ∼30–35 °C), reducing the electricity required to reach the target operating temperature (∼50 °C). The urine itself is not directly heated. A 50% heat recovery efficiency is assumed based on the typical HRV performance.^32^ This reduces the electricity demand for the concentration unit process from 600 to 300 Wh/L of raw urine. The drying process, which occurs separately at a centralized facility, is also modeled to demand 300 Wh/L of concentrated urine.

The second scenario, semicentralized treatment (S2—basement-level), is similar to the one examined by Aliahmad et al.^22^ As illustrated in Figure S6 in the Supporting Information, urine is collected, stabilized, and concentrated in the basement of each building. Similar to the first scenario, the urine concentrate is stored onsite before being transported to the final drying facility. The basement contains a 1 m^3^ tank, which takes approximately 142 days to fill at an estimated inflow of 0.007 m^3^/day of concentrate, resulting in about 2.6 tank emptyings per year. Each transport trip covers a 20 km round trip to the drying facility, with each trip moving around 20,200 kg km; the total transport amounts to 416 kg km per capita per year, comparable to S1. Once dried and pelletized, the urine-derived fertilizer is delivered to a local farm to replace synthetic fertilizers, as in the other two scenarios. Mass balance calculations are detailed in Table S13 of the Supporting Information. This scenario differs from the first primarily in its urine collection system, requiring more extensive piping to transport urine from individual toilets to the basement-level treatment unit. The concentration unit process in this configuration is modeled as vacuum distillation, with energy recovery via integrated heat exchangers. This mechanism provides internal heat exchange loops that recover energy from outgoing vapor to preheat incoming urine. At this intermediate scale, we assume a thermal recovery efficiency of 60–70% based on the practical performance of air-to-air heat exchangers and small-scale heat pumps commonly used in residential applications. This assumption aligns with findings from domestic wastewater heat recovery studies, such as Wehbi et al.,^33^ which report typical recovery rates in the range of 50–60%. Consequently, each of the unit processes, the concentration process and the final drying process, requires 200 Wh/L of urine.

In contrast to the other two scenarios, the third scenario, centralized treatment (S3—centralized-level), is entirely centralized and does not involve any concentration within the buildings but requires acidification for urine stabilization. As illustrated in Figure S10 in the Supporting Information, urine is collected and stabilized in the basement, similar to the second scenario; however, rather than being concentrated on site, it is transported via a sewer network over a distance of 10 km (the same distance assumed in the other scenarios) to a centralized facility, where it undergoes concentration, drying, and pelletization. This approach requires additional piping from the basement to a pumping station, followed by conveyance through the sewer network to the treatment facility. In terms of energy requirements, this scenario is the most energy-efficient, with the potential to recover up to 85% of the thermal energy. As in the basement configuration, the centralized concentration is also modeled as vacuum distillation with a full mechanical vapor recompression, enabling more efficient reuse of latent heat. To parametrize the energy demand and recovery efficiency, we refer to vendor data from KLC Cleanwater GmbH (2021)^34^ as an illustrative example of commercially available evaporator systems. These systems maximize heat reuse by compressing and recycling vapor, significantly reducing the demand for an external energy input.^35^ We do not assume the use of any specific proprietary unit but use these data to reflect plausible energy recovery levels in high-efficiency thermal concentration technologies. Based on KLC’s published specifications, up to 85% energy recovery is achievable; we adopt this figure to represent a best-case scenario, yielding a net electricity demand of 90 Wh/L of urine for each of the unit processes, the concentration process, and the final drying process.

While the final drying facility is the same across all scenarios, the net electricity required per liter of urine differs due to variations in the moisture content and thermal characteristics of the incoming concentrate, which are determined by the upstream concentration method.^12,36^ In S1 (toilet-level), the decentralized convective evaporation system has a lower dehydration efficiency, resulting in a wetter concentrate being transported to the centralized drying facility. This requires more energy for the final drying. In contrast, S2 (basement-level) uses a semicentralized distillation system with an integrated heat exchange, producing a more concentrated and drier product, which reduces the energy needed during the final drying step. In S3 (centralized-level), both the concentration and drying occur within an integrated vacuum evaporator using mechanical vapor recompression. This system recovers latent heat and operates as a continuous energy-optimized process. Based on vendor data (KLC Cleanwater GmbH, 2021), we assume up to 85% energy recovery, resulting in the lowest electricity demand. Therefore, although the same drying facility is used, the net electricity demand per liter of treated urine at the drying stage varies: 300 Wh/L in S1, 200 Wh/L in S2, and 90 Wh/L in S3, reflecting differences in the upstream moisture content and energy recovery.

### 2.2 Life Cycle Assessment Framework

#### 2.2.1 Goal and Scope Definition

This study adheres to the standardized life cycle assessment (LCA) methodology outlined in the ISO 14040/14044 framework. This methodology is designed to evaluate and quantify the potential environmental impact of a product or service throughout its entire lifecycle, encompassing raw material extraction, production, use, and end-of-life disposal, across various impact categories.

The primary objective of this LCA is to compare the environmental performance of three different urine recycling scenarios outlined in Section 2.1. The results aim to inform decision-makers, urban planners, and sanitation engineers about the trade-offs associated with decentralized, semicentralized, and centralized approaches to urine recycling. This information supports evidence-based planning for sustainable wastewater management in urban contexts. Using a consistent mass balance and a clearly defined functional unit (the treatment of one person’s annual urine excretion), this LCA examines whether the treatment location affects environmental impacts and identifies which configuration offers the most sustainable option for urine recycling and nutrient recovery. To ensure comparability across scenarios, fixed thermal energy recovery rates were applied based on the design of each configuration. Specifically, we assumed energy recovery rates of 50% for the toilet-level (S1), 60–70% for the basement-level (S2), and 85% for centralized treatment (S3). These values were used to estimate the net energy demand for the urine concentration and drying in each scenario. However, the modeling does not account for how energy demand varies with the treatment scale within a given configuration. Literature and vendor data (e.g., KLC Cleanwater GmbH^34^) suggest that the energy demand for distillation decreases with increasing throughput, particularly up to ∼500 L/h (∼10,600 PE/day), beyond which additional gains are marginal. As a result, the centralized scenario may be even more energy-efficient at larger scales than our assessment reflects.

Two primary LCA approaches exist: attributional (ALCA) and consequential (CLCA). Each serves a distinct purpose and is designed to answer different types of questions regarding the environmental performance of products or services. ALCA functions as an environmental accounting tool, estimating the share of the global environmental burden attributable to a specific product, i.e., how much of the global footprint can be assigned to the product under study. It assumes that the sum of environmental burdens from all final consumption activities equals the total anthropogenic impact.^27,37^ In the case of multifunctionality, where multiple valuable coproducts are produced, ALCA applies allocation methods to partition the impacts among outputs based on predefined criteria.^38^ CLCA, on the other hand, evaluates changes in the global environmental impact caused by decisions or interventions. It considers indirect market effects and system-wide consequences, i.e., how the global footprint is affected by the production and utilization of a product.^27,39^ In cases of coproduction, CLCA avoids allocation by assigning all impacts to the primary product and accounting for the avoided burden of the substituted coproducts.^29,40^ Despite the broader system perspective of CLCA, most published LCA studies still favor the attributional approach, with reviews indicating that 94% of examined papers adopted this method.^41^ The debate over the choice between ALCA and CLCA remains among the most prominent in the LCA community, particularly in relation to multifunctionality and the implications for decision-making.^42^ A key methodological distinction is that ALCA (cutoff system model) typically relies on average data, while CLCA utilizes marginal data to reflect system-level changes.^43^ This LCA study adopts a consequential approach, as the substitution of synthetic fertilizers with urine-derived alternatives aligns with the CLCA framework. However, this study also has a secondary objective: to investigate how the choice of modeling approach, consequential versus cutoff system models, impacts the study’s results, conclusions, and their interpretation for decision-makers.

The three scenarios examined in this study maintain consistent system boundaries in terms of which unit processes are included or excluded. While some of these processes are shared across scenarios, others are unique to individual scenarios; e.g., the sewer network is present only in the centralized scenario (S3). In general, the system boundary begins with the collection of urine, either through direct transport from the urine-diverting toilet to the treatment unit or via a pumping system through the sewer network. The urine then undergoes stabilization, concentration, final drying, and pelletization to produce a solid urine-based fertilizer, which is assumed to replace conventional synthetic fertilizers. It should be noted that the potential impacts on the downstream wastewater treatment plant, such as reduced hydraulic or nutrient load due to urine diversion, are not taken into account in this study.

#### 2.2.2 Life Cycle Inventory

The life cycle inventory (LCI) structure is based on the mapping material, energy, and emission flows within the system. The boundary conditions for each scenario were established through round table discussions involving coauthors and developers of urine recycling systems. Utilizing these established parameters, we developed the corresponding LCI, which encompasses a wide array of processes for each scenario and features a mass balance that assesses the inputs and outputs for each unit process. This includes collection systems (such as piping), sewer infrastructure (including piping, excavation, and backfilling), and operation of the treatment unit (covering chemical and energy consumption). Additionally, the LCI models the production of urine-based fertilizers and the replacement of synthetic fertilizers. The material used for the system’s construction has not been accounted for due to a lack of data on some scenarios. The Ecoinvent v3.8 consequential database (marginal inputs) was used for the foreground and background systems. It should be noted that while the Ecoinvent consequential model identifies marginal suppliers consistently across sectors, its precision varies. Marginal mixes for electricity are based on dispatch modeling and long-term projections, whereas for many materials (e.g., polypropylene pipes, gravel, steel) and transport services, the marginal suppliers are determined from broader market assumptions. These assumptions may not fully capture national- or sector-specific dynamics and thus introduce a greater uncertainty for infrastructure components than for energy use. Detailed procedures for establishing the LCIs are provided in the Supporting Information, and information regarding the composition of the marginal electricity and fertilizer market is found in Section 1.5 of the Supporting Information.

The urine dehydration technology assessed in this work has been demonstrated at a pilot scale and has shown proof of concept and feasibility under controlled conditions.^12,24^ Scaling up to centralized systems with energy recovery remains conceptual, relying on performance extrapolations from smaller scale data. Accordingly, our energy and mass balance assumptions are based on a combination of experimental pilot data and engineering-scale modeling.

#### 2.2.3 Life Cycle Impact Assessment

Our assessment used the ReCiPe 2016 method, explicitly utilizing the Midpoint version alongside Simapro software for modeling. We selected four impact categories that were considered most significant for our analysis; the rest of the impact categories are shown in Table S14 in the Supporting Information. These categories include global warming potential (GWP) expressed in kg CO_2_-equivalent, acidification in kg SO_2_-equivalent, freshwater eutrophication in kg P-equivalent, and marine eutrophication in kg N-equivalent. In addition to these environmental indicators, we applied the cumulative energy demand (CED) method to quantify the total primary energy consumed across the life cycle of the urine recycling system, reported in megajoules (MJ). This method estimates the total amount of primary energy, both renewable and nonrenewable, required to deliver the system’s function. It includes direct energy use (e.g., electricity for urine evaporation) as well as indirect energy inputs (e.g., energy used to manufacture equipment or transport materials). While CED does not reflect the environmental impact on its own, it serves as a complementary indicator by capturing the overall energy intensity of each recycling system. This is particularly valuable for comparing the resource efficiency of different treatment configurations.

#### 2.2.4 Sensitivity Analysis

Sensitivity analysis is a crucial method used in LCA studies to evaluate the robustness of the results. The results of these analyses provide insights into how variations in key parameters can influence not only the overall environmental assessment but also the conclusions drawn and their interpretations for stakeholders. Our previous study, Aliahmad et al.,^22^ identified several parameters within the urine recycling system that influenced the environmental impact. For instance, assuming 5% NH_3_ emission from the urine concentrator instead of no emissions leads to a significant increase in the acidification potential. Similarly, substituting sulfuric acid for citric acid as the stabilizing agent nearly halved the GWP. Another key finding was that applying 600 Wh/L of urine for the concentration without energy recovery increased GWP by almost 50%. Because these parameters are integral to unit processes that are common across all three treatment scenarios in this study, we assume the trends remain consistent and do not retest them here.

Instead, this LCA focuses on new sensitivity parameters specific to this study as well as one additional energy-related parameter for broader applicability. The first set of analyses evaluates the impact of the location of the final drying facility, which is assumed to be 10 km from the buildings in the baseline scenario. In particular, we examine how variations in the sewer network length affect the environmental performance of the centralized scenario (S3), identifying thresholds beyond which this configuration may become environmentally unsustainable. We also assess whether relocating the drying facility influences the decentralized (S1) and semicentralized (S2) scenarios by reducing the transport distance for the urine concentrate. Although sulfuric acid was previously shown to reduce GWP, a second sensitivity analysis will explore what combination of configuration adjustments (including stabilizing chemical choice and treatment location) could result in net-negative impacts across all assessed impact categories. Finally, to examine the influence of regional energy supply characteristics, we replaced the Swedish marginal electricity mix (baseline) with the EU marginal mix. This allows the assessment of result robustness in regions with a higher average grid carbon intensity. These sensitivity analyses help identify how changes in the infrastructure, chemical use, and electricity supply affect the three treatment configurations and whether they alter the comparative ranking.

## 3 Results and Discussion

### 3.1 Environmental Impact of Different Treatment Locations

The primary research question that this study aimed to address is how the location of urine treatment affects the environmental performance of urine recycling systems. The net characterized results using the consequential system model shown in Table 1 indicate that the basement-level scenario has the most favorable environmental performance across all investigated impact categories, outperforming both the toilet-level and centralized treatment configurations. Notably, the basement scenario has a Global Warming Potential (GWP) of 8 kg CO_2_-eq/capita y, which is approximately half the GWP of the other two scenarios. For a more straightforward interpretation, Figure 2 illustrates the contributions of individual unit processes to the overall impact in each scenario. It is important to note that some unit processes are unique to specific configurations; for example, the sewer network is present only in the centralized scenario. The figure also highlights the net environmental savings (negative emissions) from substituting the synthetic fertilizer with a urine-derived fertilizer, which are not explicitly detailed in Table 1, as they are integrated into the net results shown. All three scenarios are assumed to recover an equal quantity of nutrients and, therefore, yield identical climate benefits from fertilizer substitution, contributing −26 kg CO_2_-eq/capita y to the net GWP in each case.

**Table 1:** Characterized Life Cycle Assessment Results for Three Urine Recycling Scenarios with Different Treatment Locations, Calculated Using the ReCiPe Method (ReCiPe-LCA)^a^

impact category	unit	toilet (S1)	basement (S2)	centralized (S3)
global warming	kg CO_2_ eq/capita y	17	8	16
acidification	kg SO_2_ eq/capita y	6.7 × 10^–2^	5.0 × 10^–2^	8.0 × 10^–2^
eutrophication (P)	kg P eq/capita y	1.9 × 10^–3^	1.0 × 10^–3^	5.1 × 10^–3^
eutrophication (N)	kg N eq/capita y	3.0 × 10^–3^	3.0 × 10^–3^	3.2 × 10^–3^

a Results are reported per capita per year (capita y). All scenarios include synthetic fertilizer substitution benefits, which are integrated into the net impact values shown.

**Figure 2:**
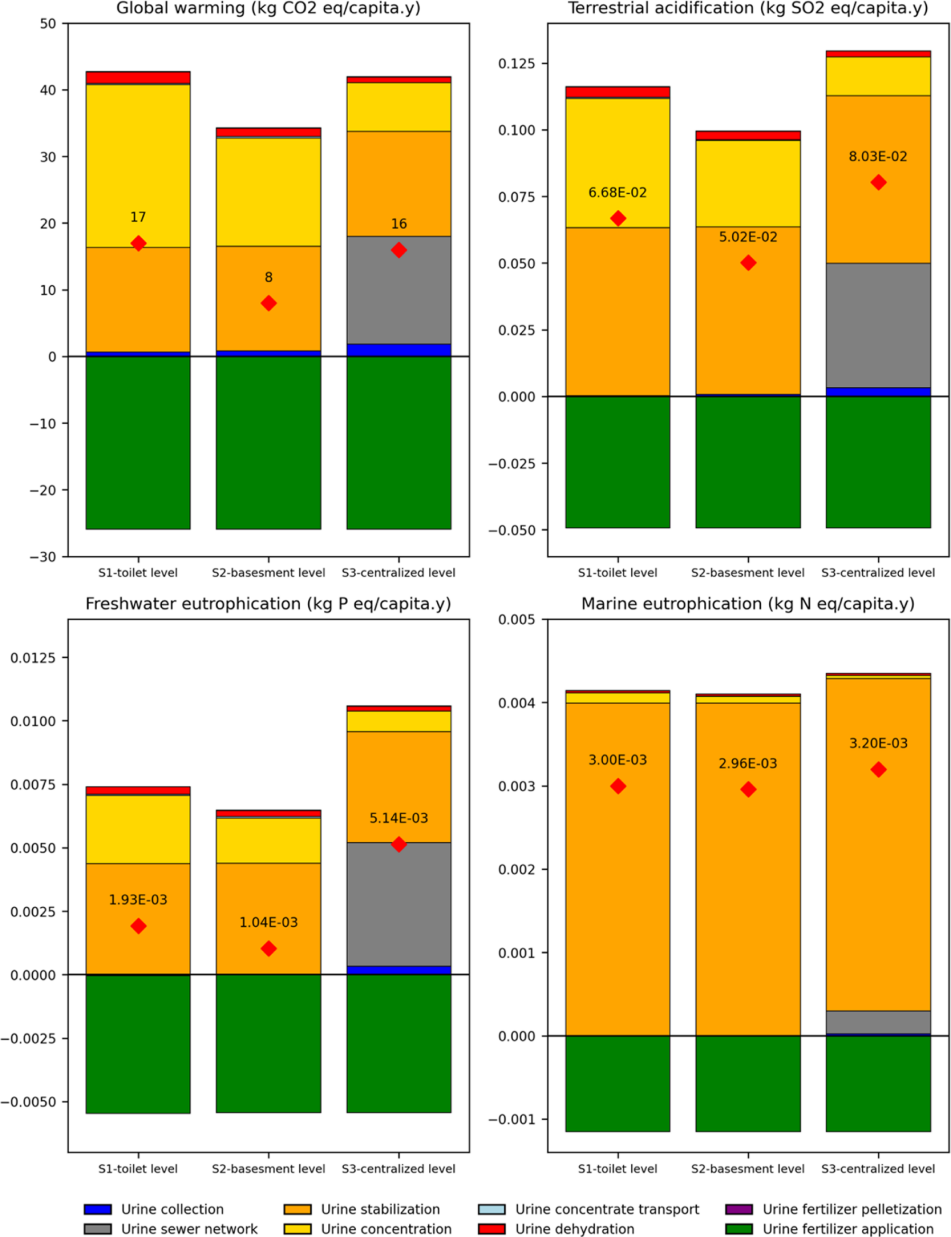
Net environmental impacts of the three urine recycling scenarios (S1: toilet-level, S2: basement-level, and S3: centralized-level), evaluated using the ReCiPe method. Results are presented across four impact categories: global warming (kg CO_2_-eq), terrestrial acidification (kg SO_2_-eq), freshwater eutrophication (kg P-eq), and marine eutrophication (kg N-eq), normalized per capita per year (PE/y). Colored bars represent contributions from individual unit processes, while red diamonds indicate net impact values after accounting for avoided impacts from the synthetic fertilizer substitution.

### 3.2 Environmental Hotspots across the Three Scenarios

#### 3.2.1 Decentralized Household Treatment (S1—Toilet-Level)

The first scenario (S1—toilet-level) exhibited the highest GWP among the three configurations, with a net impact of 17 kg of CO_2_-eq/capita y. The primary hotspot in this scenario is the urine concentration unit process, which accounts for 24 kg of CO_2_-eq/capita y. The second major contributor is urine stabilization, with a GWP of 16 kg of CO_2_-eq/capita y, largely due to the use of citric acid. Because the same amount of citric acid is applied per liter of urine in all three scenarios, the stabilization-related GWP remains consistent across them. Other unit processes, including urine collection, dehydration, and pelletization, contribute minimally, with respective values of 0.64, 1.7, and 0.05 kg CO_2_-eq/capita y. The transport of the urine concentrate (411 kg km/capita y) contributes 0.22 kg CO_2_-eq/capita y to GWP, which is small compared to the concentration and stabilization processes. Results across other impact categories, including acidification and eutrophication, show similarly higher values compared with the basement scenario. These are primarily attributed to the higher energy consumption associated with toilet-level treatment. A detailed breakdown of environmental contributions by unit processes is provided in Figure S12 in the Supporting Information.

#### 3.2.2 Semicentralized Treatment (S2—Basement-Level System)

The second scenario (S2—basement-level) results in a GWP of 8.0 kg CO_2_-CO_2_-equivalent/capita y, which is 53% lower than the toilet-level scenario. This reduction primarily arises from the decreased energy consumption in the concentration unit process, which consumes approximately 83 kWh/capita y and contributes 16 kg CO_2_-equivalent/capita y, a 32% reduction compared to S1. The second largest contributor to GWP is the urine stabilization unit process, which, as in the other scenarios, relies on citric acid dosing and contributes around 16 kg of CO_2_-equivalent/capita y. The remaining unit processes of urine collection, dehydration, and pelletization contribute less to GWP, with respective values of 0.8, 1.2, and 0.05 kg of CO_2_-equivalent/capita y. Notably, urine collection in this scenario has a 25% higher GWP than in the toilet-level scenario, attributed to the need for additional piping to convey urine from each toilet to a shared basement-level tank, unlike in S1, where each toilet is directly connected to a nearby treatment unit placed in the same room. Transport-related GWP is similar to S1, reflecting comparable annual transport work (416 kg km/capita y), despite fewer trips per year from a larger tank capacity. Across all investigated impact categories, the basement scenario consistently shows a more favorable environmental performance. A detailed breakdown of contributions by unit processes is shown in Figure S13 in the Supporting Information.

#### 3.2.3 Centralized Treatment (S3—Centralized-Level System)

The third scenario (S3—centralized-level) has a GWP of 16 kg CO_2_-equivalent/capita y, nearly identical to the toilet-level scenario and about 50% higher than the basement-level scenario. Although this system is the most energy-efficient in the concentration unit process, consuming only 37 kWh/capita y and contributing 7.3 kg CO_2_-equivalent/capita y (a reduction of 55% and 70% compared to the toilet and basement scenarios, respectively), its overall GWP is high. This is primarily due to the emissions associated with the sewer network, which contributes approximately 16 kg CO_2_-equivalent/capita y to the total impact. A breakdown of the sewer unit process shows that the main contributors to its GWP are the polypropylene pipes (10.51 kg of CO_2_-eq/capita year) and the gravel used for trench bedding and backfilling (4.99 kg of CO_2_-eq/capita year). Other contributors, such as excavation with hydraulic diggers (0.58 kg CO_2_-eq/capita year), chromium steel components for pumps (0.05 kg CO_2_-eq/capita year), and transport (0.05 kg CO_2_-eq/capita year), are comparatively minor, see Figure S16 in the Supporting Information. In this scenario, the urine is pumped through a dedicated sewer network from the basement of each building to a centralized treatment plant. This contrasts with the other two systems, where urine concentrate is directly transported by a vehicle. The stabilization unit process using citric acid also has a notable GWP estimated at 16 kg of CO_2_-equivalent/capita y. Other unit processes, such as urine collection, dehydration, and pelletization, contribute minimal amounts to GWP, with respective values of 1.85, 0.84, and 0.05 kg of CO_2_-equivalent/capita y. Although marginal, the urine collection process in this scenario has a 65% and 57% higher GWP than that of the first and second scenarios. This increase stems from the requirement for additional piping infrastructure to convey urine from each toilet to the basement and then through a trunk sewer line to a central pumping station. In contrast, the other systems carry out urine pretreatment locally within the buildings and only transport the concentrate. It is worth noting that the high sewer-related GWP in this configuration is partly due to the assumption of entirely new trench installation. While the largest share of emissions comes from the polypropylene pipes, which would still be required, reusing existing utility trenches could avoid most excavation and gravel bedding impacts, lowering sewer-related GWP by roughly one-third. Such a change could reduce the carbon footprints of the centralized configuration and make it more competitive with that of the basement-level system. Across the other impact categories, the centralized scenario performs poorly compared with the other systems, particularly for acidification and freshwater eutrophication, again largely due to the sewer infrastructure needs. A detailed breakdown of contributions by unit processes is provided in Figure S14 in the Supporting Information.

#### 3.2.4 Cumulative Energy Demand

The cumulative energy demand (CED) using the consequential model for the three urine recycling scenarios is shown in Figure 3. Among them, the second scenario (S2-basement level) has the lowest overall energy demand at 516 MJ/capita·y (≈143 kWh/capita y, given 1 kWh = 3.6 MJ). Notably, this scenario has the lowest energy demand, even when the thermal energy recovery is excluded from the analysis. To contextualize these values, consider that a typical European household consumes approximately 1.3 tons of oil equivalent (toe) annually (≈15,119 kWh, given one toe = 11,630 kWh).^44^ In comparison, treating one person’s annual urine production in Scenario 2 requires only 0.8% of this total annual energy consumption. Relative to Sweden’s national average electricity use, approximately 12,000 kWh per capita per year across all sectors, Scenario 2 represents about 1% of a person’s annual electricity footprint.^45^ For further perspective, 516 MJ/PE/y is roughly equivalent to 15 L of gasoline per year (1 L ≈ 34 MJ), enough to fuel an average passenger car for around 200 km/y. This comparison illustrates the relatively modest energy demand required to process urine using acid stabilization and evaporation in a basement-level urine recycling system, particularly when paired with thermal energy recovery systems.

**Figure 3:**
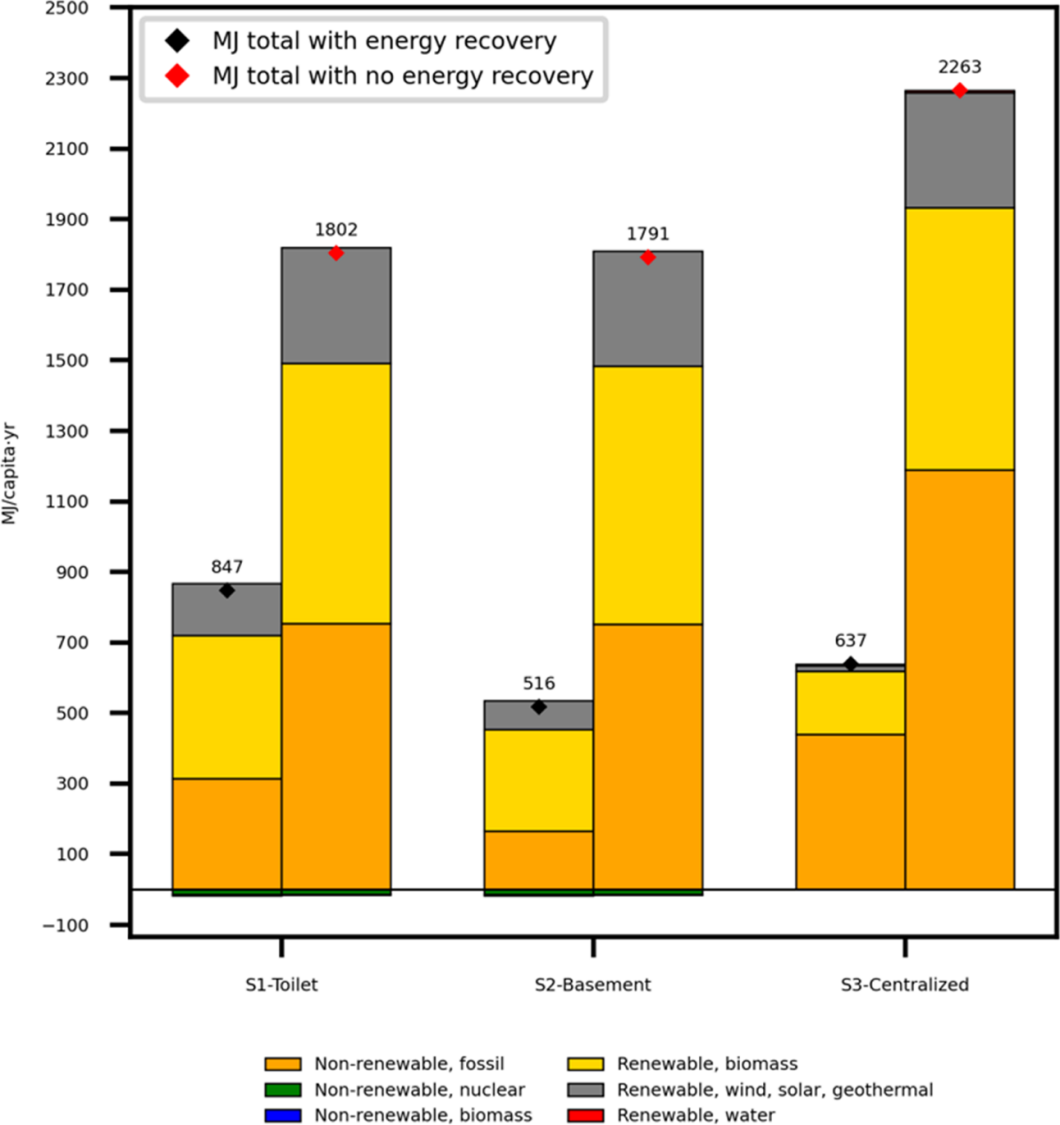
Cumulative energy demand (CED) per capita per year (capita/y) for the three urine recycling scenarios (S1: toilet-level, S2: basement-level, and S3: centralized-level). Results are disaggregated by the energy source and presented with and without heat energy recovery. Red diamonds indicate CED values without energy recovery, while black diamonds show values with energy recovery.

The CED per unit process is illustrated in Figure S15 in the Supporting Information, highlighting that the urine concentration (largely due to electricity use) and stabilization (due to citric acid production) significantly contribute to CED in the first two scenarios, whereas the sewer network is the dominant contributor in the third scenario. Notably, a urine-based fertilizer shows a negative CED, indicating that it offsets more energy use than it consumes. This credit arises from avoiding the energy-intensive production of synthetic fertilizers through the Haber–Bosch process and the extraction of mineral phosphate fertilizers. However, CED does not account for the additional energy that would have been required to remove urine-derived nitrogen and phosphorus from conventional wastewater treatment plants.

### 3.3 Impact of Life Cycle Assessment System Models on the Global Warming Potential Results

As stated in Section 3.2.1, ALCA is based on average data, whereas CLCA models are based on marginal suppliers who can adjust production in response to changes in demand and market requirements.^29^ Initially, when this LCA was first conducted, all inputs were modeled using a consequential system perspective. Under this model, the first scenario (S1—Toilet) exhibited the highest GWP of 17 kg of CO_2_ equiv/capita y, which was comparable to the centralized scenario (S3) and 50% higher than the basement-level scenario (S2). However, when the system modeling approach was switched to a cutoff model under ALCA, the results changed markedly. In the ALCA model, the first scenario (S1—Toilet) now resulted in a net negative GWP of −8 kg of CO_2_ equiv/capita y. This value was comparable to the second scenario (S2—basement) and lower than the third scenario (S3—centralized), as illustrated in Figure 4. These discrepancies primarily arise from two methodological factors: the use of average and marginal factors and the inclusion of substitution in ALCA.^46^ In the cutoff ALCA model, average emission factors are applied, which may, in certain instances, result in lower calculated emissions compared to the marginal approach, particularly in contexts like Sweden, where low-carbon renewable energy sources dominate the national energy mix. As a result, the climate impact of electricity use in processes, such as the urine concentration, is relatively small. In contrast, the CLCA model assumes that the increased electricity demand is met by marginal energy suppliers, which typically are fossil-fuel-based, leading to higher associated emissions.

**Figure 4:**
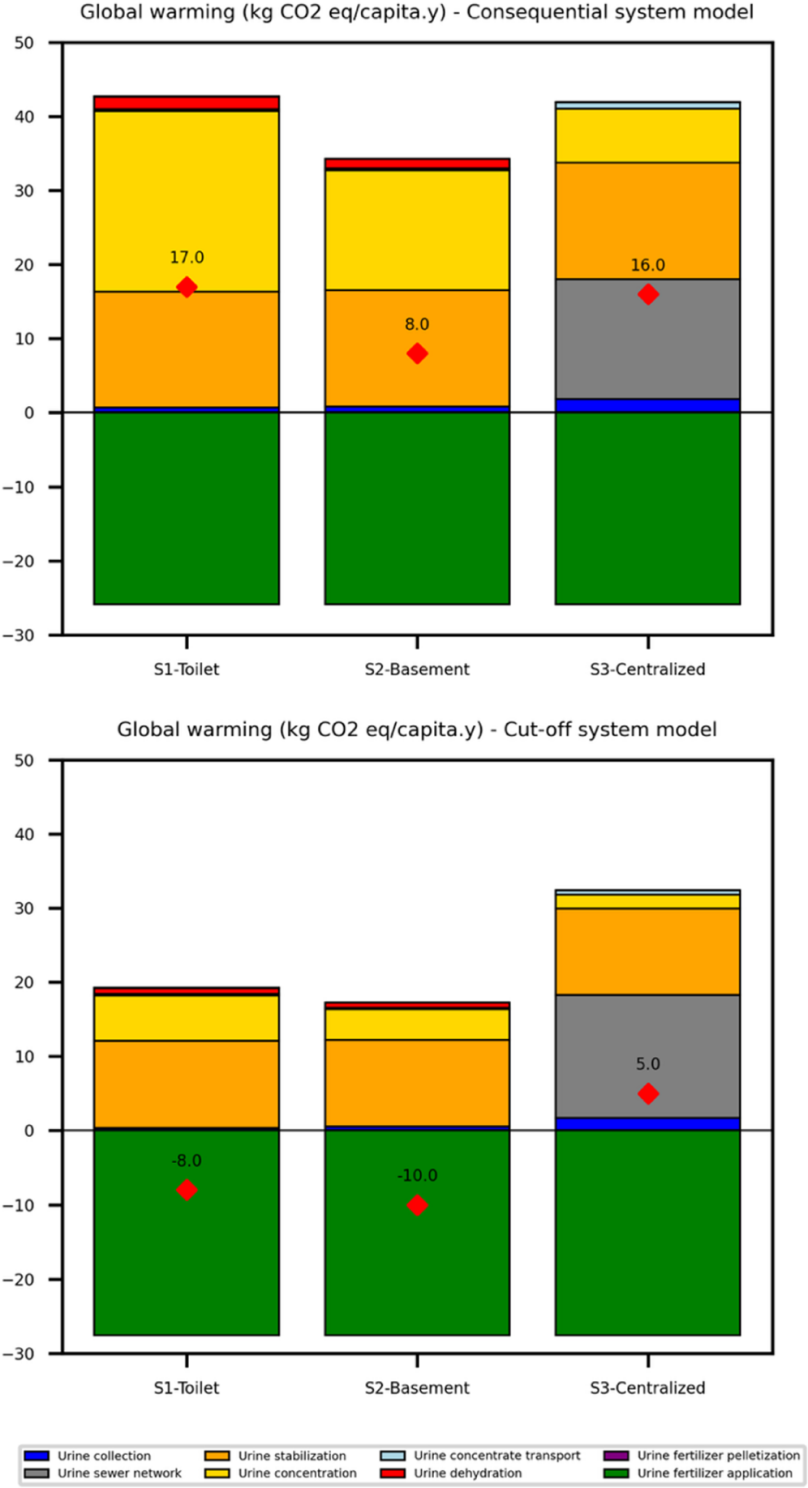
Impact of the LCA system modeling approach (cutoff versus consequential) on the Global Warming Potential (GWP) results for three urine recycling scenarios (S1—toilet, S2—basement, and S3—centralized). The top panel presents GWP outcomes using a consequential system model, while the bottom panel shows results under a cutoff attributional model. Bars indicate the contribution of individual unit processes, while red diamonds mark the total net GWP (kg CO_2_-eq per capita per year).

The second key factor contributing to the discrepancy and the net negative GWP values in the first and second scenarios is the use of substitution (i.e., accounting for the replacement of the synthetic fertilizer with a urine-derived fertilizer) within ALCA. One of the most persistent critiques of LCA studies in wastewater treatment is the lack of methodological transparency, particularly concerning the choice of the LCA framework. Many studies do not disclose whether they use attributional or consequential LCA.^47^ For example, Heimersson et al.^48^ reviewed 62 wastewater-related LCA studies and found that most did not explicitly state the type of LCA employed. Additionally, many studies appear to adopt hybrid approaches, such as avoiding allocation through substitution in ALCA and/or modeling-substituted products using average data in CLCA. Although substitution is mathematically feasible in ALCA, its application often lacks an internal logic when based on average data. ALCA is inherently designed to reflect an accounting perspective, which contradicts the substitution method that benefits from avoided burdens outside the physical system. ALCA provides a representation of the current status quo and the actual physical burdens,^49^ offering a snapshot of static impacts without considering future effects.^50^

Multiple studies recommend that substitution is more suitable within a CLCA framework and should be avoided in ALCA.^27,49,51,52^ As noted in Section 2.2.1, the two LCA approaches are designed to answer fundamentally different questions.^29^ Hence, merging divergent methodological elements can introduce inconsistencies and result in uncertain and even misleading results.^53^ However, these recommendations are often overlooked in practice, as most ALCAs appear to use substitution to resolve multifunctionality problems.^42^ Applying substitution with average data can lead to the underestimation of environmental burdens, as it credits systems for avoided impacts that do not, in reality, occur.^49^ Hence, the LCA results may neither reflect the true share of the global environmental load attributable to the studied system nor accurately capture the changes that would result from the system’s introduction.^47^

This inconsistency is evident in our study. When substitution was applied in ALCA (Figure 4), the net GWP values for all three scenarios decreased significantly, resulting in negative values for the first two scenarios. However, this outcome hinges on problematic assumptions. For example, if a region’s nitrogen fertilizer mix includes both unconstrained synthetic fertilizer (e.g., urea) and constrained organic fertilizer (e.g., manure from local livestock farms), claiming that the urine-based fertilizer offsets the entire nitrogen mix is inaccurate. Manure, as a constrained byproduct of livestock production, cannot simply be scaled up or down. Even if it is not applied locally, it will likely be utilized elsewhere. Thus, only unconstrained inputs, such as urea, can be legitimately displaced by a urine-derived fertilizer. Even studies that tolerate substitution in ALCA argue that, if applied, it should be based on unconstrained marginal technologies that can respond to market dynamics.^54^

### 3.4 Sensitivity Analysis Results

The results of the sensitivity analysis are listed in Figure 5. The first analysis examined the effect of reducing the transport distance to the final drying plant from 10 km to 5 km on the GWP across the three urine recycling scenarios. This relocation had a marginal effect on the first two scenarios but a significant effect on the third. This disparity stems from the relative contribution of the sewer network to the third scenario’s overall GWP. Specifically, reducing the transport distance to 5 km led to a GWP reduction of only 1% for the first two scenarios, from 16.8 to 16.7 for S1 and 8.4 to 8.3 kg CO_2_-eq/capita y for S2, respectively. The minor change is attributable to a small reduction in emissions from the concentrate transport, from 0.22 to 0.11 kg CO_2_-eq/capita y. In contrast, for S3, the shorter sewer distance significantly reduced GWP, from 16 to 8.2 kg CO_2_-eq/capita y, representing a 49% decrease. The decline is due to the decrease in sewer network GWP, which dropped from 16.17 to 8.34 kg CO_2_-eq/capita y. Thus, the net GWP of the third scenario became comparable to that of the basement-level scenario. Nevertheless, S3 still exhibited higher impacts in other categories, as described in the Supporting Information.

**Figure 5:**
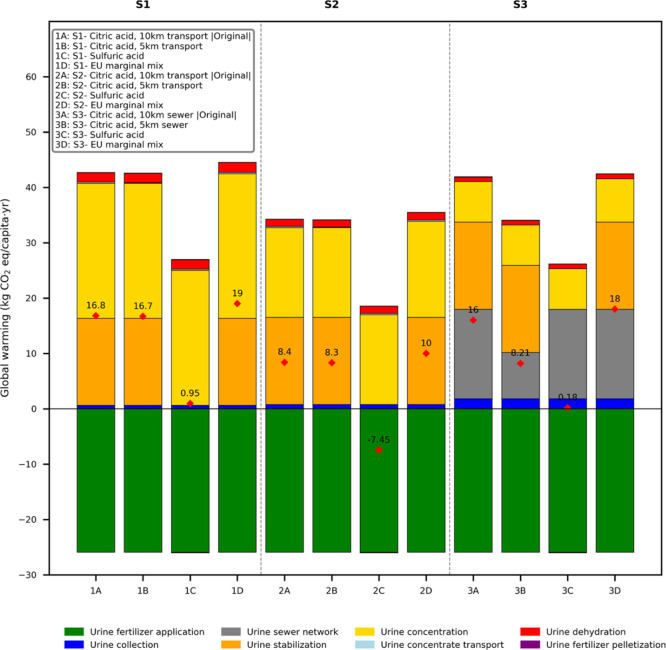
Impact of sensitivity analysis scenarios on the global warming potential (GWP) of the three urine recycling scenarios (S1—toilet, S2—basement, S3—centralized). The analysis includes two parameters: (i) reducing transport or sewer distances from 10 km to 5 km (scenarios S1, S2, S3), and (ii) substituting citric acid with 1.36 g/L sulfuric acid (scenarios S1, S2, S3). The red diamonds indicate net GWP (kg CO_2_-eq/capita y).

The second sensitivity analysis explored alternative chemical inputs and energy recovery assumptions to identify the most environmentally favorable configuration capable of achieving net-negative impacts across all categories. The literature suggests that sulfuric acid has a lower GWP than citric acid, as it is often produced as a byproduct in industrial processes such as copper smelting and crude oil desulfurization. Substituting citric acid with 1.36 g of sulfuric acid per liter of urine led to a notable decrease in GWP across all scenarios, resulting in reductions of 94%, 190%, and 99% for S1, S2, and S3, respectively. This translates to a GWP reduction of 16.8–0.95 (S1), 8.4 to −7.45 (S2), and 16–0.18 kg CO_2_-eq/capita y (S3), as shown in Figure 5. Among the three scenarios, S2 (basement-level treatment) emerged as the most environmentally effective configuration with a net negative GWP of −7.45 kg CO_2_-eq/capita y, owing to the combined effects of sulfuric acid use and 70% heat energy recovery. To explore the robustness of this finding, an additional test examined the minimum energy recovery threshold required for S2 to remain carbon negative. The results showed that this configuration could sustain as little as 52% energy recovery and still maintain a net-negative carbon footprint.

Finally, replacing the Swedish marginal electricity mix with the EU marginal mix increased the net GWP to 19 kg of CO_2_-eq/capita y for S1, 10 for S2, and 18 for S3. The absolute increase was the largest for the electricity-intensive S1 and smallest for S3. Importantly, the ranking remained unchanged (S2 < S3 ≈ S1), indicating that the comparative conclusions are robust across regions with a higher grid carbon intensity.

### 3.5 Interpretation for Decision Making

This LCA study indicates that the second scenario (S2—basement-level treatment) offers the most favorable environmental profile among the three configurations analyzed. Across all impact categories and modeling approaches, the basement scenario consistently demonstrates the lowest environmental burdens. However, it is essential to note that the material used for the construction of the urine recycling system, including treatment units, storage tanks, and ancillary infrastructure, was not accounted for in this study due to incomplete data for some scenarios. This omission means that the results cannot be interpreted as fully comprehensive, and further work is needed to incorporate these life cycle stages for a more definitive conclusion. In practice, the types and quantities of construction materials are likely to differ across the three scales. For example, the toilet-level system (S1) would require a compact but oversized heat pump to handle intermittent household flows, whereas the basement-level system (S2) would integrate a dedicated heat exchanger sized for multiapartment use. The centralized system (S3) replaces building-level evaporation with a large-scale vapor evaporator, using mechanical vapor recompression. Storage requirements also differ: S1 relies on small frequent-emptying containers; S2 uses intermediate-scale tanks to buffer multibuilding flows; and S3 includes large-scale centralized storage to manage peaks from a wider catchment. These differences could influence the environmental profile if construction and replacement impacts were included. Although adding construction materials would increase the total GWP for all scenarios, scenario 2 might require less total material than scenario 1 (fewer, larger units instead of many smaller ones) and scenario 3 (less extensive facility, storage, and sewer infrastructure). Therefore, while accounting for construction impacts would raise the overall impacts, it is unlikely to change the ranking order, and it could actually strengthen the favorable performance of scenario 2.

The most environmentally optimal configuration for S2 involves replacing citric acid with sulfuric acid as the stabilizing agent, which results in a net negative environmental profile. Despite the environmental advantages of sulfuric acid, several practical challenges may limit its application. Its use requires following stringent safety protocols during storage, transport, and handling, particularly if used near end-users, such as household or toilet-level treatment units. Furthermore, although sulfuric acid can be produced as an industrial byproduct, its supply chain is currently tied to fossil fuel-intensive processes. This dependence conflicts with broader sustainability objectives aimed at shifting to fossil-free systems and raises concerns about its long-term availability.^55^ The baseline assumption for energy recovery in the basement scenario was set at 70%, but sensitivity analysis revealed that the system remains carbon negative, even at a reduced recovery rate of 52%, suggesting that this configuration can remain robust under varying operational efficiencies.

The GWP results obtained from the two modeling systems (consequential vs attributional cutoff) varied considerably, highlighting the importance of methodological transparency to decision-making. These discrepancies are particularly pronounced when substitution is incorporated within ALCA. For stakeholders seeking a static snapshot of a product’s status or environmental profile, specifically the share of the global burden attributable to that product, the attributional (cutoff) model is generally recommended. The attributional cutoff model allocates impacts to the product’s upstream consumption and enforces the “polluter pays” principle.^56^ It considers only the system’s direct physical inputs and outputs, where recyclable materials are “cut-off” from the system, treated as burden-free, while all waste-related impacts are wholly attributed to the producer. In this model, byproducts may either be allocated proportionally (e.g., by weight or cost) or removed without burden if recognized as recyclable. In contrast, consequential LCA (CLCA) analyzes the broader environmental implications of decisions, particularly those that influence supply chains and market dynamics. CLCA is appropriate when decision-makers aim to understand how introducing a product affects the global environmental burden. Instead of allocation, CLCA employs substitution: if a byproduct can substitute for another product in the market, environmental credits are assigned for the avoided production. In this study, for instance, a urine-derived fertilizer is assumed to substitute a synthetic fertilizer, and the producer gains credit for avoiding production. Importantly, CLCA emphasizes the role of “unconstrained/marginal” suppliers of synthetic fertilizer who are capable of adjusting production in response to shifts in the market demand.^29^

The system models also differ in the type of data drawn from the database Ecoinvent, in this case. For example, the urine recycling system involves the use of plastic for urine collection, and the associated environmental impacts vary, depending on the system model selected from the database. In both attributional and consequential models, virgin plastic carries the full burden of its production. However, when recycled plastic is used in the cutoff model, it is considered burden-free, with only recycling impacts accounted for, meaning no credits are granted to the producer. In contrast, the consequential model treats recycled plastic as a substitute for virgin plastic, awarding credits for avoiding virgin production. An increase in the demand for virgin plastic triggers marginal suppliers to boost production, which introduces additional environmental impacts. If recyclable plastic replaces other materials in this model, the producer receives credit through substitution.

The interpretation of cumulative energy demand outcomes is heavily influenced by the choice of the LCA modeling approach. The cutoff model reflects the average national energy mix and offers a snapshot of the system’s current environmental impact, while the consequential system model focuses on marginal energy sources activated by the increased demand, providing a more dynamic perspective that is better suited for evaluating the effects of scaling or systemic changes.^57^ In the consequential model, the primary energy supply from marginal producers is shaped by an incremental demand, which is typically met in the short term by fossil-fuel-based sources such as gas turbines or coal-fired units. As such, this modeling approach might provide a more accurate representation of the real implications associated with implementing new technologies, including urine recycling sanitation systems.^58^ While the impact of a urine recycling system may be minimal at the individual level, its nationwide implementation can significantly alter electricity demand profiles. For example, if urine recycling were to replace conventional wastewater treatment across an entire city, introducing millions of new electric appliances, such as heaters, dryers, and pumps, the electricity grid would be forced to adjust. Under these conditions, the marginal energy mix becomes increasingly critical. Thus, the consequential model is advantageous for policy evaluation, strategic sustainability planning, and forecasting environmental impacts associated with the large-scale adoption of new systems.

The ongoing debate between ALCA and CLCA, particularly regarding the handling of multifunctionality and the appropriateness of applying substitution within the ALCA, remains a complex and unsettled issue. This LCA study does not seek to determine which approach is the most suitable. Rather, it emphasizes the importance of transparency in disclosing the type of LCA conducted and the system modeling choices made, as such clarity is essential to ensure that decision-makers correctly interpret results. Fundamentally, ALCA and CLCA are designed to answer different questions, and therefore, providing conflicting results without specifying the underlying methodology can lead to confusion and misinformed decisions, undermining the replicability of these LCAs and hindering their use by other practitioners. Just as it is crucial to clearly define the functional unit, it is equally important to specify the type of LCA being performed, the approach taken to resolve multifunctionality, and whether substitution (if applied) is based on average or marginal data. Drawing conclusions or comparing results across divergent LCA types without proper context adds to the ambiguity and contributes to the ongoing discord within the LCA community.

Beyond the environmental metrics, real-world implementation should also account for practical and contextual constraints.^59^ Labor needs, for instance, are not captured in this LCA but can strongly influence the feasibility. The toilet-level scenario (S1) is expected to be the most labor-intensive due to the frequent handling of small storage units and decentralized maintenance. The basement-level scenario (S2) centralizes these tasks at the building scale, reducing labor requirements, while the centralized scenario (S3) is likely to require the least day-to-day labor, as most processes occur at a single facility. While S2 demonstrated the best environmental performance, local conditions for implementation may favor other options. Reusing existing sewer trenches, for example, could lower the footprint of S3, making it more competitive. Where sewer installation is impractical, basement- or toilet-level systems may be preferable, and in existing buildings with technical barriers to basement installation, S1 may be the better retrofit choice. For new constructions, however, S2 remains the most advantageous. Ultimately, by combining a robust environmental assessment with the consideration of labor, infrastructure, and site constraints and maintaining transparency in LCA modeling, urine recycling can be strategically implemented as a scalable low-impact alternative to conventional sanitation.

## Supplementary Material



## Notes

LCI data, including wastewater characteristics, mass and energy balances, system boundaries and assumptions, wastewater characteristics, layout of each scenario, data used for modeling urine collection; agriculture application of urine NPK pellets; urine drying; urine stabilization and concentration; and sewer network; NPK application, characterized life cycle assessment results, GWP, cumulative energy demand, and schematic diagram of the primary unit process (PDF)
