# Supplementary material for: Comparative Environmental Assessment of Three Urine Recycling Scenarios: Influence of Treatment Configurations and Life Cycle Modeling Approaches
Source: Environ Sci Technol. 2025 Sep 24;59(39):21160–73. doi: 10.1021/acs.est.5c09248 (PMC12509321; doi:10.1021/acs.est.5c09248)
Supplement: Supplementary file 1 [file es5c09248_si_001.pdf]

SUPPORTING INFORMATION

Comparative environmental assessment of three urine recycling scenarios: Influence of treatment configurations and life cycle modeling approaches.

Abdulhamid Aliahmad [a], *, Prithvi Simha[a], Björn Vinnerås [a], Jennifer McConville [a]

[a] Department of Energy and Technology, Swedish University of Agricultural Sciences, Box 7032, S-75007 Uppsala, Sweden

* Corresponding author.
E-mail address: Abdulhamid.aliahmad@slu.se
Summary: 19 pages, 14 Figures, 14 Tables

Table of Contents

1. WASTEWATER CHARACTERISTICS 2

2. S1: DECENTRALIZED HOUSEHOLD TREATMENT (S1—TOILET-LEVEL)....3

1.1 URINE COLLECTION INSIDE THE BATHROOM OF EACH APARTMENT BUILDING 4
1.2 URINE PRETREATMENT INSIDE THE BATHROOM (STABILIZATION AND CONCENTRATION)....5
1.3 URINE DRYING 6
1.4 AGRICULTURE APPLICATION....6
1.5 MARGINAL ELECTRICITY MIX AND FERTILIZER....7

3. S2: SEMI-CENTRALIZED TREATMENT (S2—BASEMENT-LEVEL) 8

2.1 URINE COLLECTION INSIDE THE BASEMENT OF EACH BUILDING 8
2.2 URINE PRETREATMENT IN THE BASEMENT (STABILIZATION AND CONCENTRATION)....10
2.3 URINE DRYING 10

4. S3: CENTRALIZED TREATMENT (S3—CENTRALIZED-LEVEL) 11

3.1 URINE COLLECTION INSIDE THE BASEMENT OF EACH BUILDING AND SEWER NETWORK....11
3.2 URINE TREATMENT IN THE CENTRALIZED FACILITY....13

6. MASS BALANCE....14

5. RESULTS....16

....22

LIFE CYCLE INVENTORY

The following figures and tables present the data considered in the life cycle inventory (LCI). The data collected pertain to the three scenarios considered in the LCA: decentralized household treatment (S1—toilet-level), semi-centralized treatment (S2—basement-level), and centralized treatment (S3—centralized-level). The functional unit is the management of domestically generated urine per capita per year, including collection, treatment, and disposal/reuse.

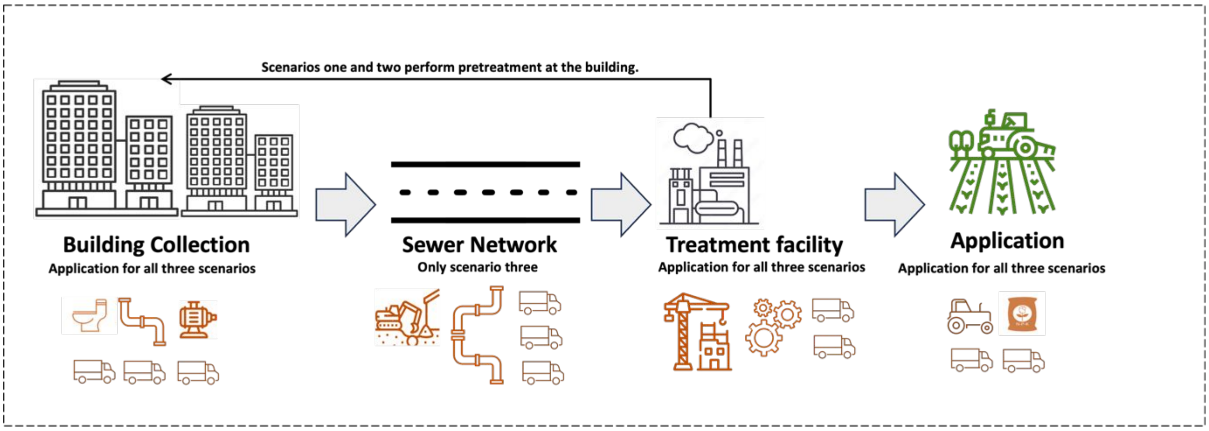

Figure S 1: Segments included in the modelling of the three scenarios. The icons represent system segments (collection, conveyance, treatment, storage, transport, and application), arrows indicate material flows between segments, and the dashed boundary outlines the study system boundary.

1. Wastewater characteristics

Table S 1: Wastewater characteristics used for modeling the urine recycling systems, the nitrogen, phosphorus, and potassium values are highlighted.

**Wastewater**			
COD	64,1	g/p.d	faeces. Jönsson et al. 2005
TS	53,1	g/p.d	faeces+toilet paper. Jönsson et al. 2005
TSS	28,76	g/p.d	faeces+toilet paper. Jönsson et al. 2005
VS	53,8	g/p.d	faeces+t.p. Jönsson et al. 2005
P-tot	0,5	g/p.d	faeces (0.5). Jönsson et al. 2005
N-tot	1,5	g/p.d	faeces (1.5). Jönsson et al. 2005
NH4-N	0,3	g/p.d	faeces (0.3). Jönsson et al. 2005
COD	8,5	g/p.d	Urine. Jönsson et al. 2005
TS	20	g/p.d	Urine. Jönsson et al. 2005
TSS	20	g/p.d	Assume TSS zero so TS = TSS
VS		g/p.d	Urine. Jönsson et al. 2005
**P-tot**	**0,9**	**g/p.d**	**Urine. Jönsson et al. 2005**

**N-tot**	**11**	**g/p.d**	**Urine. Jönsson et al. 2005**
NH4-N	10,3	g/p.d	Urine. Jönsson et al. 2005
**K**	**2**	**g/p.d**	**Urine. Jönsson et al. 2005**

Urine characteristics

COD	7521	mg/L
TS	17697	mg/L
TSS	17697	mg/L
VS	0	mg/L
P-tot	796	mg/L
N-tot	9733	mg/L
NH4-N	9114	mg/L

Urine Flow

Urine excreted	550	L/p.y	Total urine a person excrete a year (about 1,5 L/capita.d)
Urine captured	412,5	L/p.y	75% of urine is separated in the UDT
Total urine separated	141,267	L/d	multiply by 125 capita (total number of people in the 5 buildings).
Total urine separated	0,141	m3/d	Convert to m3.

2. S1: Decentralized household treatment (S1—toilet-level)

2.1 Urine collection inside the bathroom of each apartment building

75% of the collected urine is collected separately in the toilet and then flows into the concentrator, also inside the bathroom, to undergo pretreatment. The uncollected urine and the rest of the wastewater are collected inside the building and transported together from inside to outside the property using the same pipe. Then, they are transported to the wastewater treatment plant via the sewer network. See **Error! Reference source not found.** below for illustration.

- Subprocesses included: the pipe material (its manufacturing process and transportation from the provider to the site), and the toilet material.

- Subprocesses not included: Pipe installations inside the building and toilet manufacturing. It was assumed that urine flows by gravity, so no energy is required.

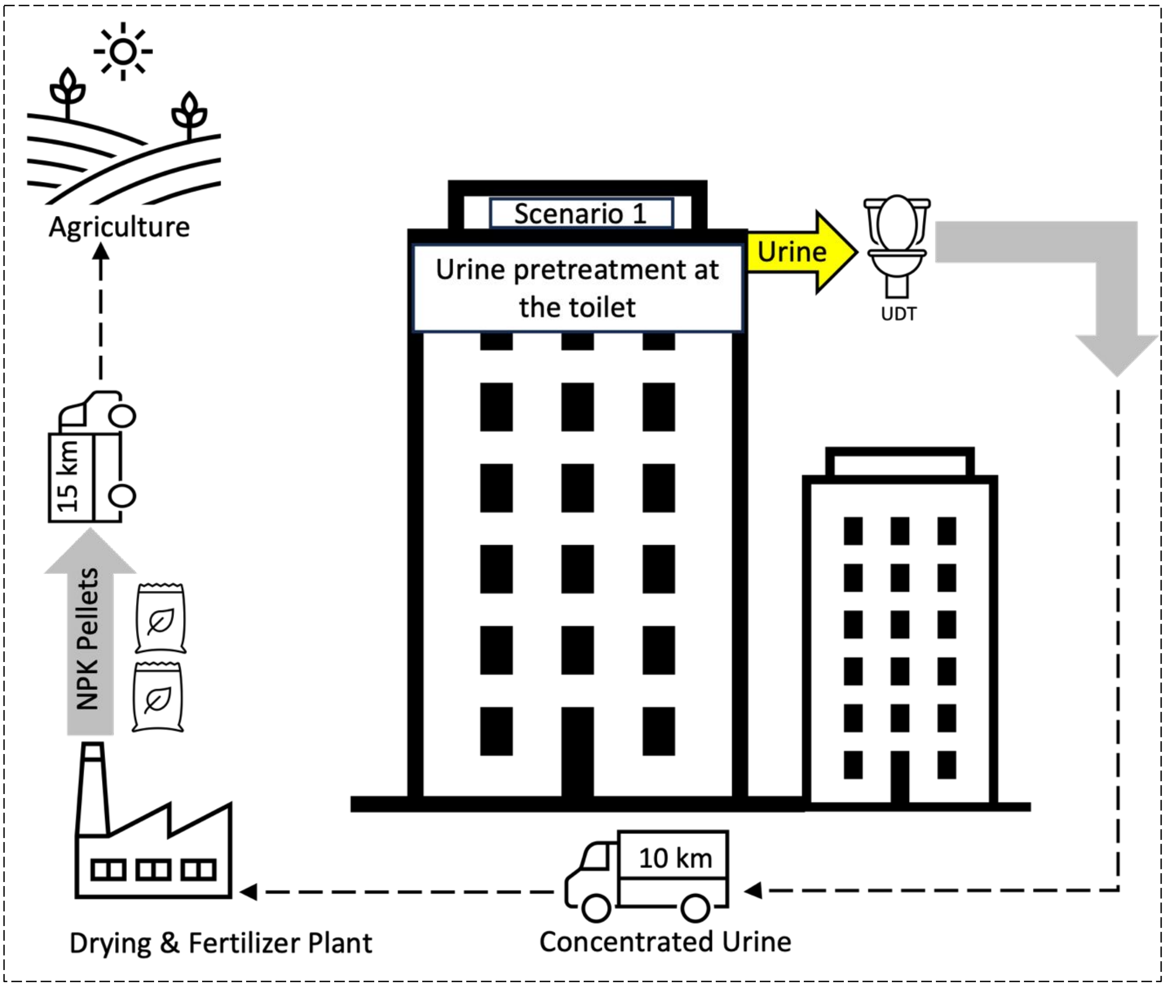

Figure S 2: The layout of the first scenario (S1– toilet-level). Urine is collected and pretreated inside the bathroom, and later, concentrated urine is transported to pelletization and NPK fertilizer production.

Table S1: Data used for modeling Urine collection inside the bathroom (per functional unit; person. year)

Concept	Units	Value	Comments	Reference
Collection inside the bathroom:				

Concept	Units	Value	Comments	Reference
Urine pipes:	kg/capita.y	0,00110	Bathroom line PP/ DN50: 2,00 mm, 3 m (per apartment), Life span: 50 years , 0,46 kg/m. Polypropylene, granulate & Extrusion, plastic pipes market for | Conseq.	
Toilet:	kg/ capita.y	0,195	Ceramic material. 24,4 kg/u. Life span: 50 years. Assumption: 1 UDT toilet per apartment. Sanitary ceramics market for | Conseq.	

2.2 Urine pretreatment inside the bathroom (stabilization and concentration).

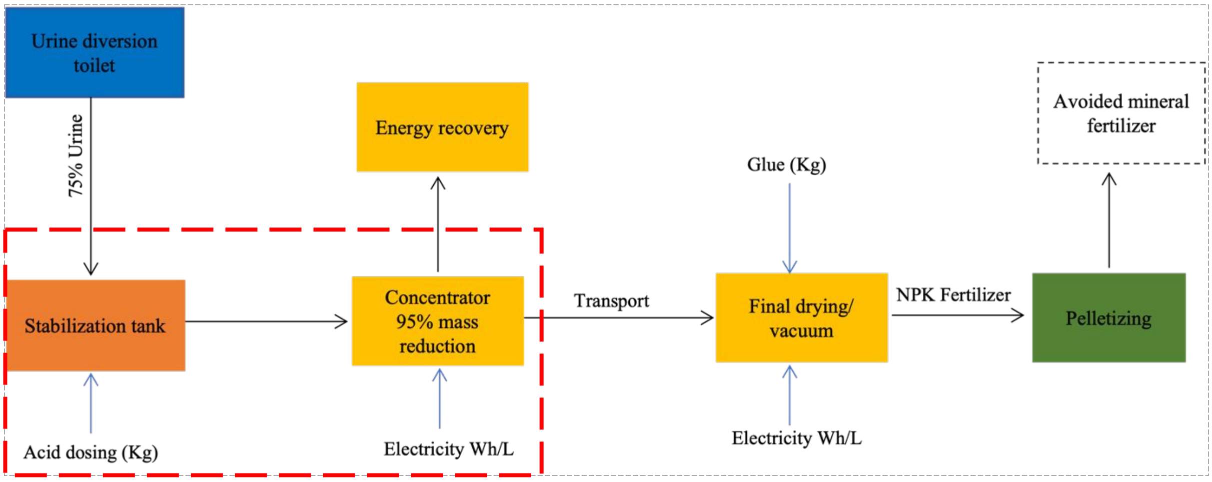

Figure S1: Scenario 1 unit process focused on pretreatment (stabilization and concentration)

Table S2: Data used for modeling urine stabilization and concentration (per functional unit; person. year)

Concept	Units	Value	Comments	Reference
Urine operation: stabilization and concentration				
**Electricity consumption:**	**kWh/ capita.y**			
Urine Concentrator	kWh/ capita.y	123,750	600 wh/L = 600 kwh/m3. Hence, it will be 247.50 kwh/p.y, however, we have 50% heat recovery so we will assume that the electricity consumption is 300 wh/ L. Electricity, low voltage {SE}| market for | Conseq.	SLU
**Chemicals:**	**kg/ capita.y**			
Citric acid $C_6H_8O_7$	kg/ capita.y	4,125	10 g/L of urine = 10 kg/m3. Assumption: local providers (20 km) and transport, lorry 3.5-7.5, Euro 6. Citric acid {RER}| market for | Conseq. Or Sulfuric acid {RER}| market for sulfuric acid | Conseq for sensitivity analysis.	

2.3 Urine drying

After urine is stabilized and concentrated, it is transported to the final drying stage, where NPK powder is produced and then pelletized for use in farmland.

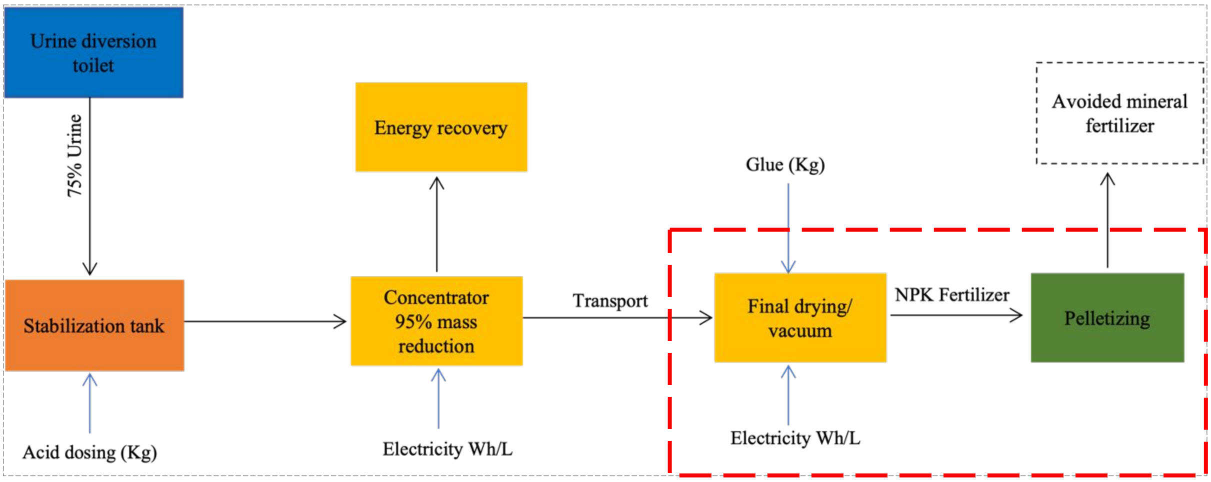

Figure S2: Scenario 1 unit process focused on final treatment (drying and pelletizing)

Table S3: Data used for modeling urine drying (per functional unit; person. year)

Concept	Units	Value	Comments	Reference
Urine drying and pelletization.				
**Electricity consumption**:	**kWh/ capita.y**			
Vacuum drying	kWh/ capita.y	6,19	600 wh/L = 600 kwh/m3. However, we have 50% heat recovery so we will assume that the electricity consumption is 300 wh/liter of concentrated urine. Electricity, low voltage {SE}| market for | Conseq.	SLU
Transport of concentrate in 1 year	kg.km/ capita.y	410,92	Transport is every 2 months, 20 km round trip. Transport, freight, lorry 3.5-7.5 metric ton, euro6 {RER}| market for transport, freight, lorry 3.5-7.5 metric ton, EURO6 | Conseq.	
Pelletization	kWh/ capita.y	0,26	Electricity, low voltage SE| market for | Conseq	
**Chemicals:**	**kg/ capita.y**			
Glue	kg/ capita.y	5,10	0.25 % of the incoming mass. Compost. Assumption market, Conseq.: local providers (50 km) and transport, lorry 3.5-7.5, Euro 6.	

2.4 Agriculture application

The urine is pretreated in the bathroom unit and then transported as a concentrated liquid to the final vacuum dryer. Then the dried powder is pelletized into NPK fertilizer that is later driven

to a local farmer to be used as an alternative to mineral fertilizer. This unit process is the same for the three scenarios and will be covered only in here.

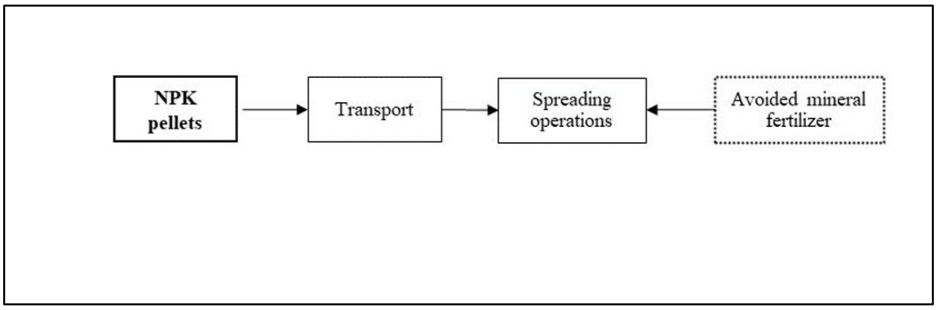

Figure S3: NPK application in farmland and mineral fertilizer substitution

Table S4: Data used for modeling agriculture application of Urine NPK pellets (per functional unit; person. year)

Concept	Units	Value	Comments	Reference
Transport	t.km/capita.y	0,391	To local farmers 15 km for all scenarios. Assumption: lorry, 3.5-7.5 metric ton, euro6	
Spreading operations	kg/ capita.y	26,04	Assumption: solid manure loading and spreading, by hydraulic loader and spreader market for | Conseq.	
Emissions to air:				(Remy and Jekel, 2010)
NH_3	kg/ capita.y	0,250	Factor : 0.063 g NH_3/g N	
N_2O	kg/capita.y	0,0497	Factor : 0.0125 g N_2O/g	
Avoided mineral fertilizer:				
N fertilizer	kg/capita.y	-3,74	Assumption: nitrogen fertilizer, as N: Inorganic nitrogen fertiliser, as N {SE}| market for inorganic nitrogen fertiliser, as N | Conseq.	
P fertilizer	kg/ capita.y	-0,77	Assumption: phosphate fertilizer, as P_2O_5 : Inorganic phosphorus fertiliser, as P2O5 {SE}| market for inorganic phosphorus fertiliser, as P2O5 | Conseq.	
K fertilizer	kg/ capita.y	-1,08	Inorganic potassium fertiliser, as K2O {SE}| market for inorganic potassium fertiliser, as K2O | Conseq.	
Avoided spreading mineral fertilizer	ha/ capita.y	-0,037	Assumption: 100 kg N/ha and fertilising by broadcaster GLO| market for | Conseq.	
Avoided emissions due to mineral fertilizer spreading:				
NH_3	kg/ capita.y	-0,046	Factor : 0.05 g NH_3/g N	
N_2O	kg/ capita.y	-0,187	Factor : 0.0125 g N_2O/g N	

2.5 Marginal electricity mix and Fertilizer

Electricity Mix: In this study, the Swedish electricity supply for the three scenarios is modeled using the consequential model for the Swedish electricity market, with the long-term marginal generation mix, which reflects Sweden's long-term marginal electricity supply. This mix includes 41.4% natural gas (combined cycle), 32.6% onshore wind (>3 MW), 23.5% wood-based electricity, and 2.46% offshore wind (1–3 MW).

Fertilizer markets: Nutrient substitution for the three scenarios is modeled using Ecoinvent consequential markets for inorganic nitrogen, phosphorus (as P_2O_5), and potassium (as K_2O) fertilizers in Sweden. The nitrogen market is mainly made up of calcium ammonium nitrate (66.7%), with additional shares from NPK (15-15-15) (26.9%) and minor sources like ammonium nitrate, phosphate, and sulfate. The phosphorus market is primarily led by NPK (15-15-15) (76.4%) and diammonium phosphate (21.7%). The potassium market is dominated by NPK (15-15-15) (74.6%) and potassium chloride (19.0%). Substitution is carried out at the nutrient level, preventing double-counting of impacts from compound fertilizers.

3. S2: Semi-centralized treatment (S2—basement-level)

3.1 Urine collection inside the basement of each building

75% of the collected urine is collected separately in the toilet and then flows by gravity to the basement for pretreatment. The uncollected urine and the rest of the wastewater are collected inside the building and transported together from inside to outside the property using the same

pipe. Then, they are transported to the wastewater treatment plant via the sewer network. See Figure S4 for illustration.

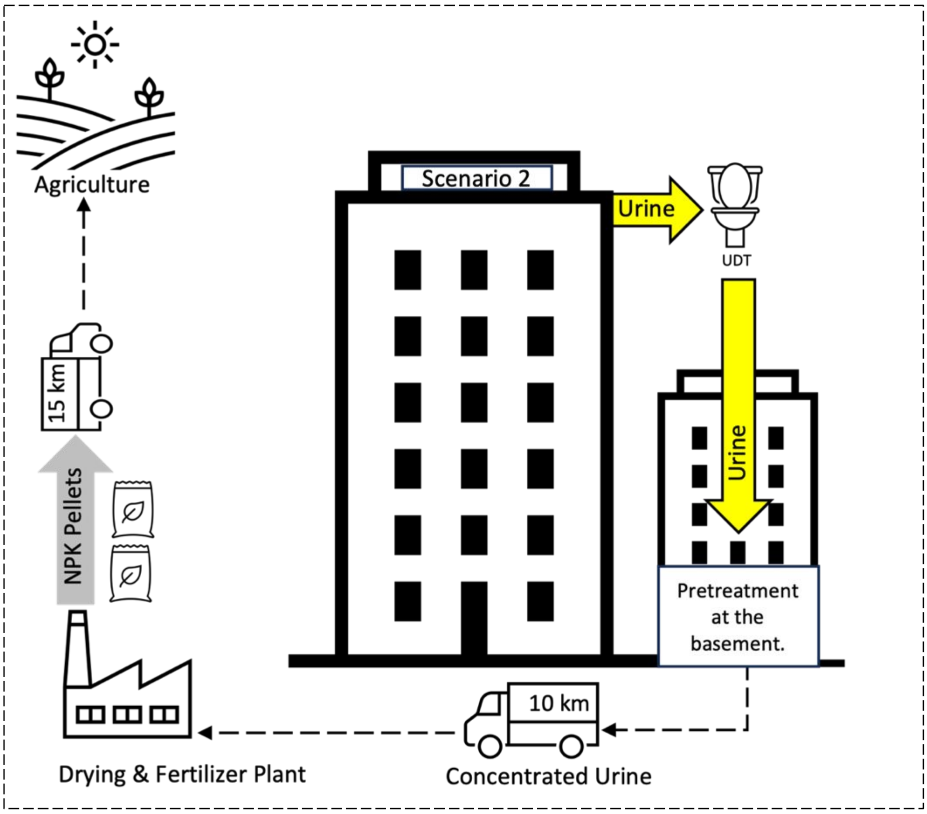

Figure S4: The layout of the second scenario (S2– basement-level). Urine is collected and pretreated inside the basement, and later, concentrated urine is transported to pelletization and NPK fertilizer production.

- Subprocesses included: the pipe material (its manufacturing process and transportation from the provider to the site), and the toilet material.

- Subprocesses not included: Pipe installations inside the building and toilet manufacturing. It was assumed that urine flows by gravity, so no energy is required.

Table S5: Data used for modeling Urine collection inside the bathroom (per functional unit; person. year)

Concept	Units	Value	Comments	Reference
Collection inside the building:				
Urine pipes	kg/ capita.y	0,065	Basement line PP/ DN75: 2,5 mm and PP/ DN50: 2,00 mm 20 m (x2 stems per building) as well as 3 m (length of pipe from toilet to down pipe) x 1 toilet/apartment x 50 apartments. Polypropylene, granulate & Extrusion, plastic pipes market for | Conseq.	
Toilet	kg/ capita.y	0,195	Ceramic material. 24,4 kg/u. Life span: 50 years. Assumption: 1 UDT toilet per apartment. Assumption: Assumption: distance 2,159 km (Switzerland, JET), lorry 3.5-7.5, Euro 6. Sanitary ceramics market for | Conseq.	

3.2 Urine pretreatment in the basement (stabilization and concentration).

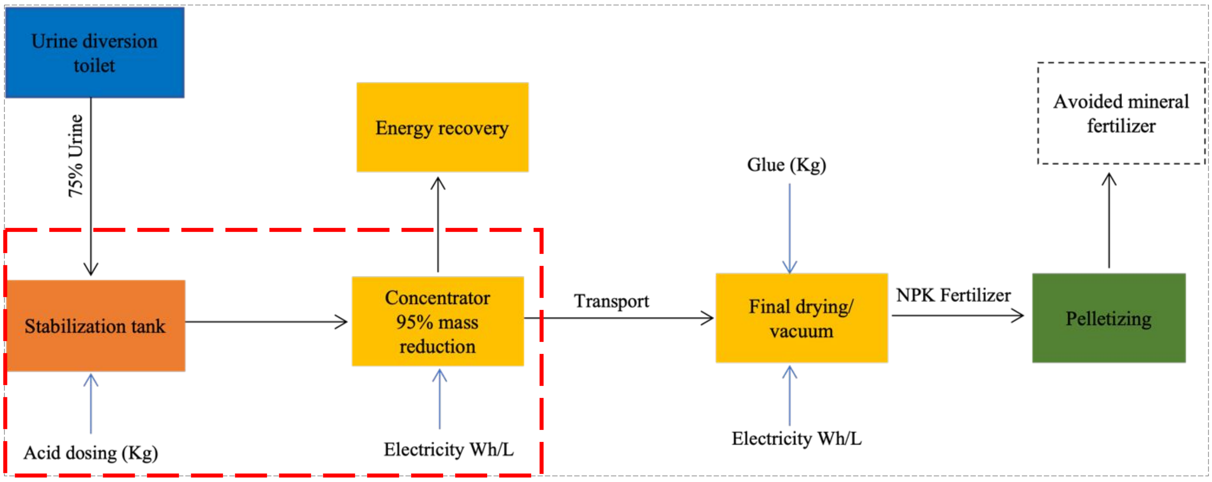

Figure S5: Scenario 2 unit process focused on pretreatment (stabilization and concentration)

Table S6: Data used for modeling urine stabilization and concentration (per functional unit; person. year)

Concept	Units	Value	Comments	Reference
Urine operation: stabilization and concentration				
**Electricity consumption:**	**kWh/ capita.y**			
Urine Concentrator	kWh/ capita.y	82,5	600 wh/L = 600 kwh/m3. Hence, it will be 247.50 kwh/p.y, however, we have about 70% heat recovery so we will assume that the electricity consumption is 200 wh/ L. Electricity, low voltage {SE}| market for | Conseq.	SLU
**Chemicals:**	**kg/ capita.y**			
Citric acid $C_6H_8O_7$	kg/ capita.y	4,125	10 g/L of urine = 10 kg/m3. Assumption: local providers (20 km) and transport, lorry 3.5-7.5, Euro 6. Citric acid {RER}| market for | Conseq. Or Sulfuric acid {RER}| market for sulfuric acid | Conseq for sensitivity analysis.	

3.3 Urine drying

After urine is stabilized and concentrated, it is transported to the final drying stage, where NPK powder is produced and then pelletized for use in farmland.

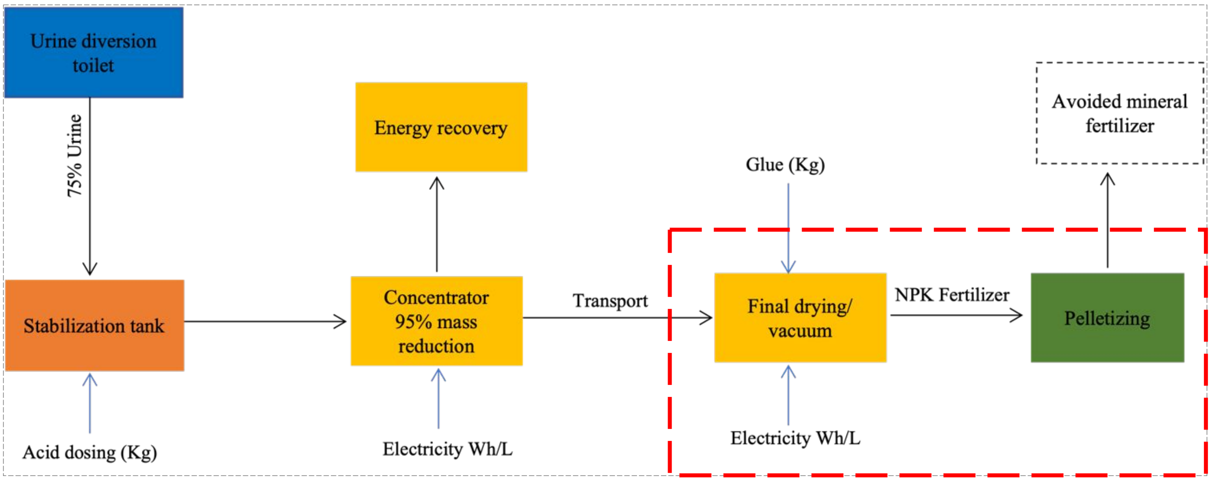

Figure S6: Scenario 2 unit process focused on final treatment (drying and pelletizing)

Table S7: Data used for modeling urine drying (per functional unit; person. year)

Concept	Units	Value	Comments	Reference
Urine drying and pelletization				
Electricity consumption:	kWh/ capita.y			
Vacuum drying	kWh/ capita.y	4,13	600 wh/L = 600 kwh/m3. However, we have 70% heat recovery so we will assume that the electricity consumption is 200 wh/liter of concentrated urine. Electricity, low voltage {SE}| market for | Conseq	SLU
Transport of concentrate in 1 year	kg.km/ capita.y	416,63	Transport is three times a year, 20 km round trip. Transport, freight, lorry 3.5-7.5 metric ton, euro6 {RER}| market for transport, freight, lorry 3.5-7.5 metric ton, EURO6 | Conseq.	
Pelletization	kWh/ capita.y	0,26	Electricity, low voltage {SE}| market for | Conseq	
Chemicals:	kg/ capita.y			
Glue	kg/ capita.y	5,10	0.25 % of the incoming mass. Compost. Assumption: local providers (50 km) and transport, lorry 3.5-7.5, Euro 6.	

4. S3: Centralized treatment (S3—centralized-level)

4.1 Urine collection inside the basement of each building and sewer network

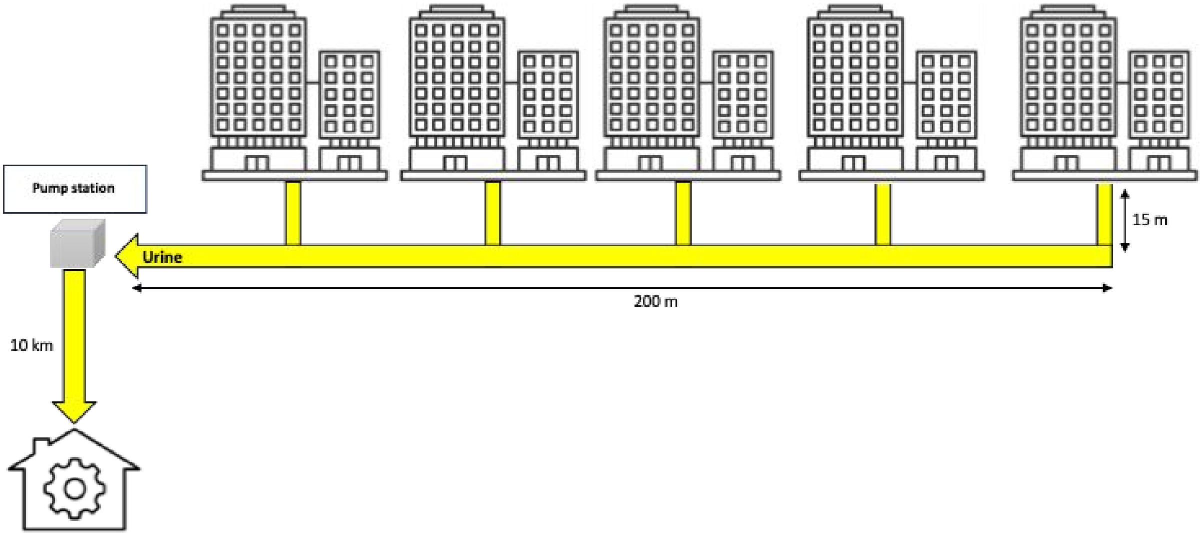

Figure S7: Scenario 3 layout regarding the collection and sewer network from buildings to the centralized treatment facility

75% of the collected urine is collected separately in the toilet and then flows by gravity to the basement for stabilization only. The urine is not concentrated, but it is transported via the sewer network to a centralized facility where it undergoes all treatment. The uncollected urine and the rest of the wastewater are collected inside the building and transported together from inside to outside the property using the same pipe. Then, they are transported to the wastewater treatment plant via the sewer network. As illustrated in Figure S8 below.

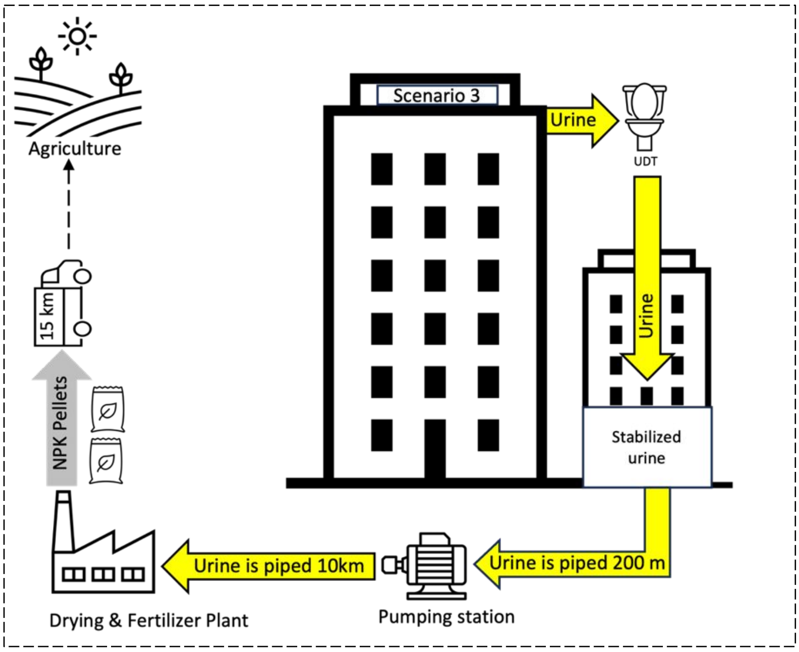

Figure S8: The layout of the third scenario (S3– centralized-level). Urine is collected and piped via a sewer network to a centralized plant for treatment, pelletization, and NPK fertilizer production.

- Subprocesses included: the pipe material (its manufacturing process and transportation from the provider to the site), and the toilet material.
- Subprocesses not included: Pipe installations inside the building and toilet manufacturing. It was assumed that urine flows by gravity, so no energy is required.

Table S8: Data used for modeling Urine collection inside the bathroom (per functional unit; person. year)

Concept	Units	Value	Comments	Reference
Collection inside the building:				
Urine pipes	kg/ capita.y	0,4817	Basement line PP/ DN75: 2,5 mm and PP/ DN50: 2,00 mm 20 m (x2 stems per building) as well as 3 m (length of pipe from toilet to down pipe) x 1 toilet/apartment x 50 apartments and transport pipe. Polypropylene, granulate & Extrusion, plastic pipes market for | Conseq.	SLU
Toilet	kg/ capita.y	0,195	Ceramic material. 24,4 kg/u. Life span: 50 years. Assumption: 1 UDT toilet per apartment. Assumption: Assumption: distance 2,159 km (Switzerland, JET), lorry 3.5-7.5, Euro 6	

Table S9: Data used for modeling the sewer network (per functional unit; person. Year)

Concept	Units	Value	Comments	Reference
Construction:				
Urine pipe	kg/capita.y	2,15E+00	PP75/9 mm, 75 m from all buildings. PP90/9 mm (trunk), 200 m connecting buildings to the pump station. PP90/9 mm (trunk), 10k sewer to the treatment station. Polypropylene, granulate market for GLO | Conseq.	SLU
Pipe transport	kg.km/capita.y	4,29E+01	Assumption: local providers (20 km)	
Pumps	kg/capita.y	8,00E-03	pumps: 1 u. 30kg/u. Pump material: stainless-steel; life span: 30 years. Steel, chromium steel 18/8 GLO| market for | Conseq.	
Excavation	m^3/capita.y	5,29E-01	The volume excavated was calculated considering the calculations of trench dimensions. Excavation, hydraulic digger RER| processing | Conseq.	(Morera et al., 2016)
Backfilling material	t/capita.y	3,58E-01	Calculations based on (Morera et al., 2016). Assumption: gravel crushed; density 1600 kg/m^3 . Gravel, crushed market for gravel, crushed | Conseq.	(Morera et al., 2016)
Operation:				
Electricity	kWh/capita.y	0,04	0,1 kWh/m3 (for pumping). Electricity, low voltage SE| market for | Conseq.	

4.2 Urine treatment in the centralized facility

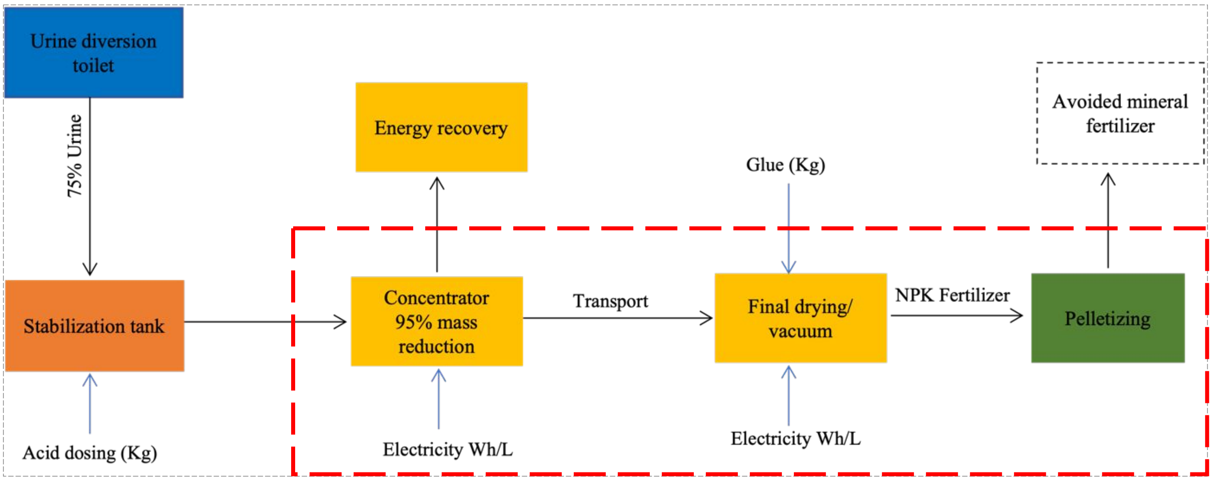

Figure S9: Scenario 3 unit process focused on final treatment (concentration, drying and pelletizing)

Table S10: Data used for modeling urine stabilization and concentration (per functional unit; person. year)

Concept	Units	Value	Comments	Reference
Urine drying and pelletization				
**Electricity consumption:**	**kWh/capita.y**			
Urine Concentrator	kWh/ capita.y	37,125	600 wh/L = 600 kwh/m3. Hence, it will be 247.50 kwh/p.y, however, we have about 85% heat recovery so we will assume that the electricity consumption is 90 wh/ Liter of concentrated urine. Electricity, low voltage SE| market for | Conseq	SLU
**Chemicals:**	**kg/ capita.y**			
Citric acid $C_6H_8O_7$	kg/ capita.y	4,125	10 g/L of urine = 10 kg/m3. Assumption: local providers (20 km) and transport, lorry 3.5-7.5, Euro 6.	
Vacuum drying	kWh/ capita.y	1,86	600 wh/L = 600 kwh/m3. However, we have 85% heat recovery so we will assume that the electricity consumption is 90 wh/l.	SLU
Pelletization	kWh/ capita.y	0,26	Electricity, low voltage SE| market for | Conseq	
**Chemicals:**	**kg/ capita.y**			
Glue	kg/ capita.y	5,10	0.25 % of the incoming mass. Compost. Assumption: local providers (50 km) and transport, lorry 3.5-7.5, Euro 6.	

5. Mass balance

Table S11: Mass balance of incoming urine in scenario 1, starting from the concentrator, where 95% of the mass is reduced, then to the final dryer, where the NPK powder is produced, and the emptying time of the tank in the bathroom unit.

**input**	**Quantity**	**Units**	**Origin**	**Quantity per capita.year**	**Units**	**Comments**
Flow in concentrator	0,141	m^3/d	Stabilization tank	0,413	$m^3/p.y$	
**output**	**Quantity**	**Units**	**Destination**	**Quantity per capita.year**	**Units**	**Comments**
[3] Flow out	0,007	m^3/d	Final/vaccum drying	0,021	$m^3/p.y$	
			-			
**input**	**Quantity**	**Units**	**Origin**	**Quantity per capita.y**	**unit**	**Comments**
[3] Flow in	0,007	m^3/d	Concentrator		$m^3/p.y$	Fully dried
Time before concentrate transport	60	days	transport every 2 months. Concentration unit is 10 L			1.130 L per person equivalent per day (PE.day), resulting in about 0.057 L of concentrate after a 95% mass reduction. The overall quantity of concentrate produced per apartment daily is 0.143 L, totaling 8.6 L over the course of 60 days, with an additional 1.5 L held in buffer to prevent overflow during emergencies or excessive inflow
Frequency of concentrate transport	6	Trips/year				
Transport of concentrate in 1 trip	8560,788	kg.km/trip	Round trip 10 km * 2			
Transport of concentrate in 1 year	51364,726	kg.km/year		410,92	kg.km/p.y	simapro

Table S12: Mass balance of incoming urine in scenario 2, starting from the concentrator, where 95% of the mass is reduced, then to the final dryer, where the NPK powder is produced, and the emptying time of the tank in the basement.

**input**	**Quantity**	**Units**	**Origin**	**Quantity per capita.year**	**Units**	**Comments**
Flow in concentrator	0,141	m^3/d	Stabilization tank	0,413	$m^3/p.y$	
**output**	**Quantity**	**Units**	**Destination**	**Quantity per capita.year**	**Units**	**Comments**
Flow out of concentrator	0,007	m^3/d	Final/vaccum drying	0,021	$m^3/p.y$	

**input**	**Quantity**	**Units**	**Origin**	**Quantity per capita.y**	**unit**	**Comments**
Flow in dryer	0,007	m^3/d	Concentrator		m^3/p.y	Fully dried
Time for full tank (1 m3)	141,58	Days	Assume a 1 m3 tank in the basement			1 m3 tank in the basement
Frequency of transport per year	2,58	Trips / year	Round trip 10 km * 2			
Transport of concentrate in 1 trip	20200	kg.km/trip	Each trip is (1000 kg * 20 km = 20,000 kg.km) 1m3 urine mass	161,60	kg.km/ p.y	
Transport of concentrate in 1 year	52078,125	kg.km/year		416,63	kg.km/ p.y	
Glue	1,783	kg/d	Commercial product	5,21	kg/p.y	0.25 % of the incoming mass
Transport Glue	35,670	kg.km/d	*Assumption*: distance 50 km	104,16	kg.km/ p.y	
**output**	**Quantity**	**Units**	**Destination**	**Quantity per capita.y**	**unit**	**Comments**
Dried mixture	8,917	kg/d	Pelletization process	26	kg/p.y	Everything in the urine
Nitrogen powder	1,361	kg/d	Pelletization process	3,97	kg/p.y	
Phosphorus powder	0,111	kg/d	Pelletization process	0,33	kg/p.y	
Pottasium powder	0,308	kg/d	Pelletization process	0,90	kg/p.y	
NPK powder	1,780	kg/d	Pelletization process	5,20	kg/p.y	Nitrogen powder + Phosphorus powder

6. Results

Table S13: Characterized life cycle assessment results for three urine recycling scenarios with different treatment locations, calculated using the ReCiPe® method (ReCiPe-LCA). Results are reported per person

equivalent per year (PE/y). All scenarios include synthetic fertilizer substitution benefits, which are integrated into the net impact values shown.

Impact category	Unit	0. Full system (S1)	0. Full system (S2)	0. Full system (S3)
**Global warming**	kg CO2 eq	17	8	16
**Stratospheric ozone depletion**	kg CFC11 eq	1,75E-05	8,84E-06	4,01E-06
**Ionizing radiation**	kBq Co-60 eq	-8,15E-01	-8,22E-01	-4,88E-01
**Ozone formation, Human health**	kg NOx eq	3,21E-02	1,36E-02	4,48E-02
**Fine particulate matter formation**	kg PM2.5 eq	2,23E-02	1,65E-02	3,48E-02
**Ozone formation, Terrestrial ecosystems**	kg NOx eq	3,25E-02	1,37E-02	4,68E-02
**Terrestrial acidification**	kg SO2 eq	6,68E-02	5,02E-02	8,03E-02
**Freshwater eutrophication**	kg P eq	1,93E-03	1,04E-03	5,14E-03
**Marine eutrophication**	kg N eq	3,00E-03	2,96E-03	3,20E-03
**Terrestrial ecotoxicity**	kg 1,4-DCB	3,15E+01	-3,87E+01	-3,08E+01
**Freshwater ecotoxicity**	kg 1,4-DCB	4,40E+00	2,54E+00	1,09E+00
**Marine ecotoxicity**	kg 1,4-DCB	5,30E+00	3,02E+00	1,30E+00
**Human carcinogenic toxicity**	kg 1,4-DCB	4,54E-01	1,52E-01	3,46E-01
**Human non-carcinogenic toxicity**	kg 1,4-DCB	8,69E+00	4,38E-02	3,22E+00
**Land use**	m2a crop eq	1,34E+01	8,55E+00	4,33E+00
**Mineral resource scarcity**	kg Cu eq	-1,84E-01	-1,87E-01	-1,06E-01
**Fossil resource scarcity**	kg oil eq	6,88E+00	3,59E+00	9,61E+00
**Water consumption**	m3	9,52E-02	7,45E-02	4,47E-01

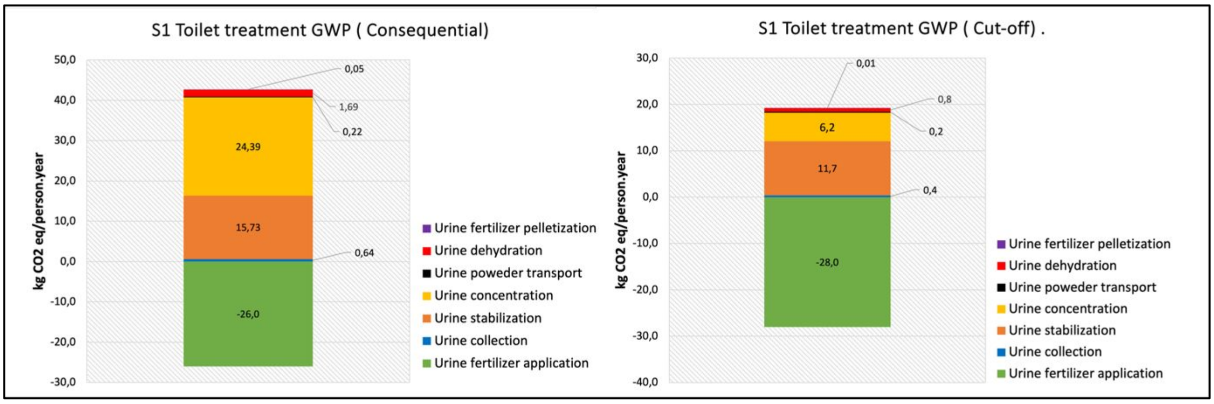

Figure S10: GWP of scenario 1 comparing two system models, consequential and cut off

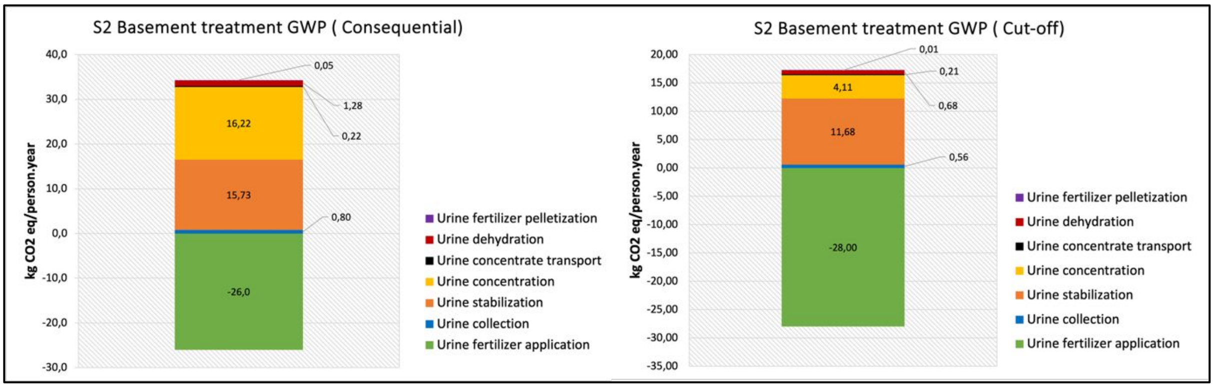

Figure S11: GWP of scenario 2 comparing two system models, consequential and cut off

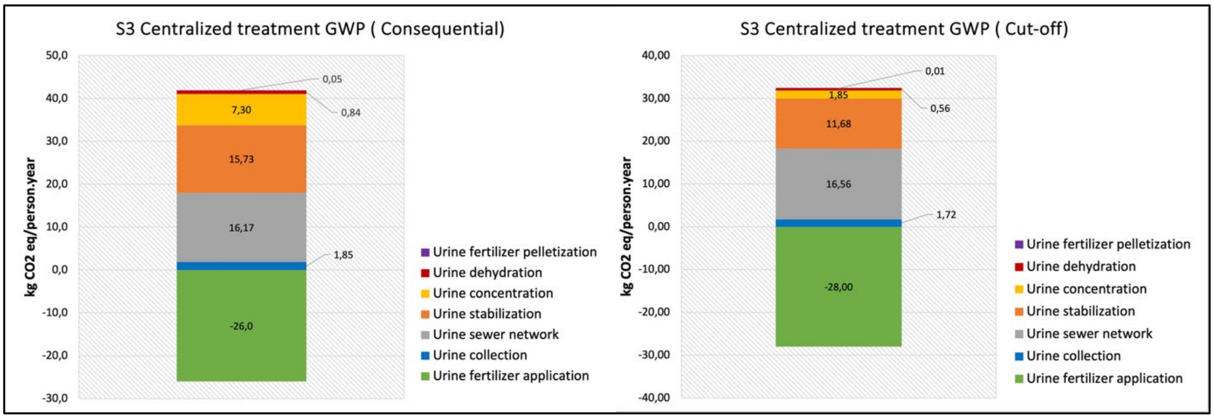

Figure S12: GWP of scenario 3 comparing two system models, consequential and cut off

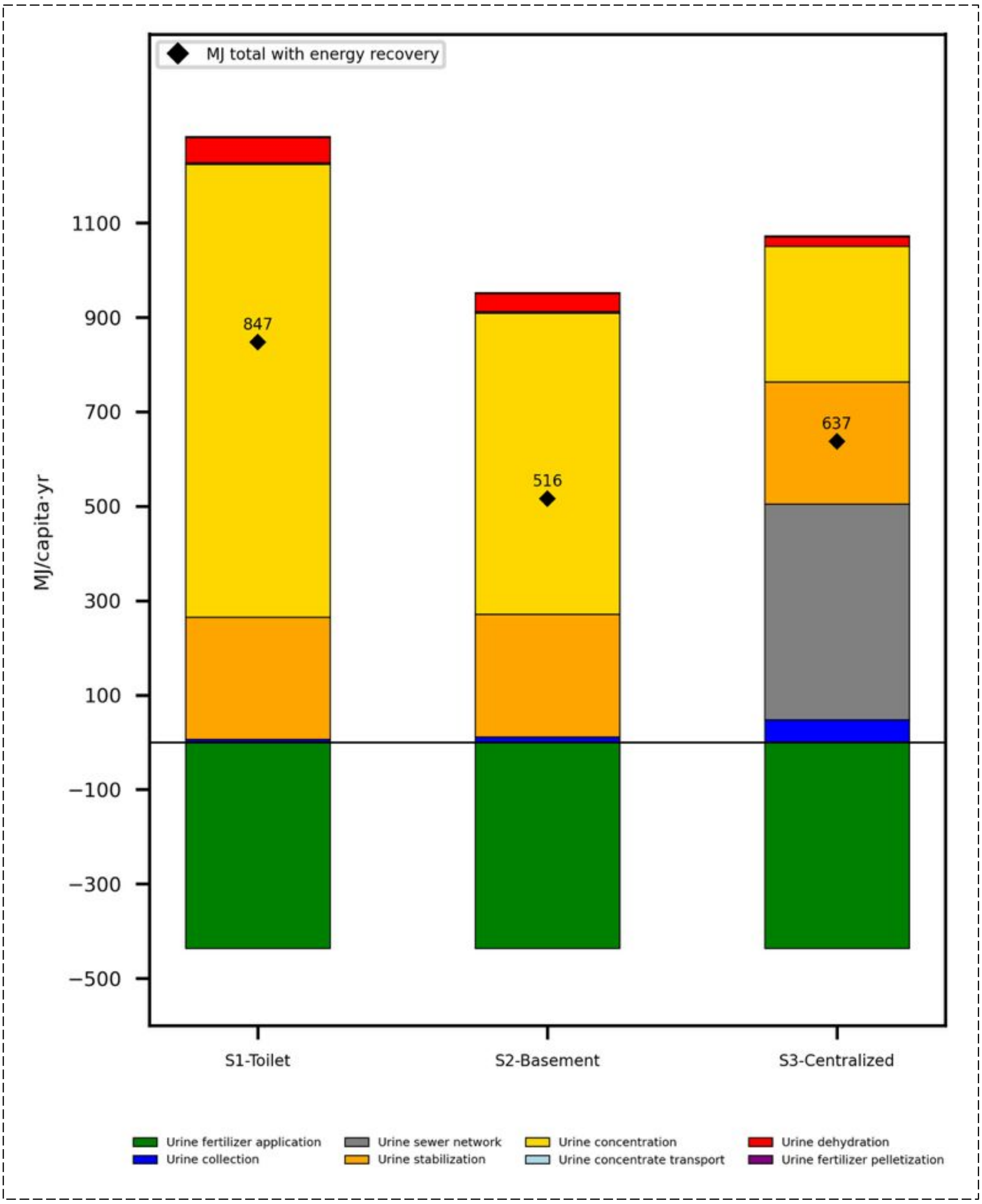

Figure S13: The cumulative energy demand per unit process.

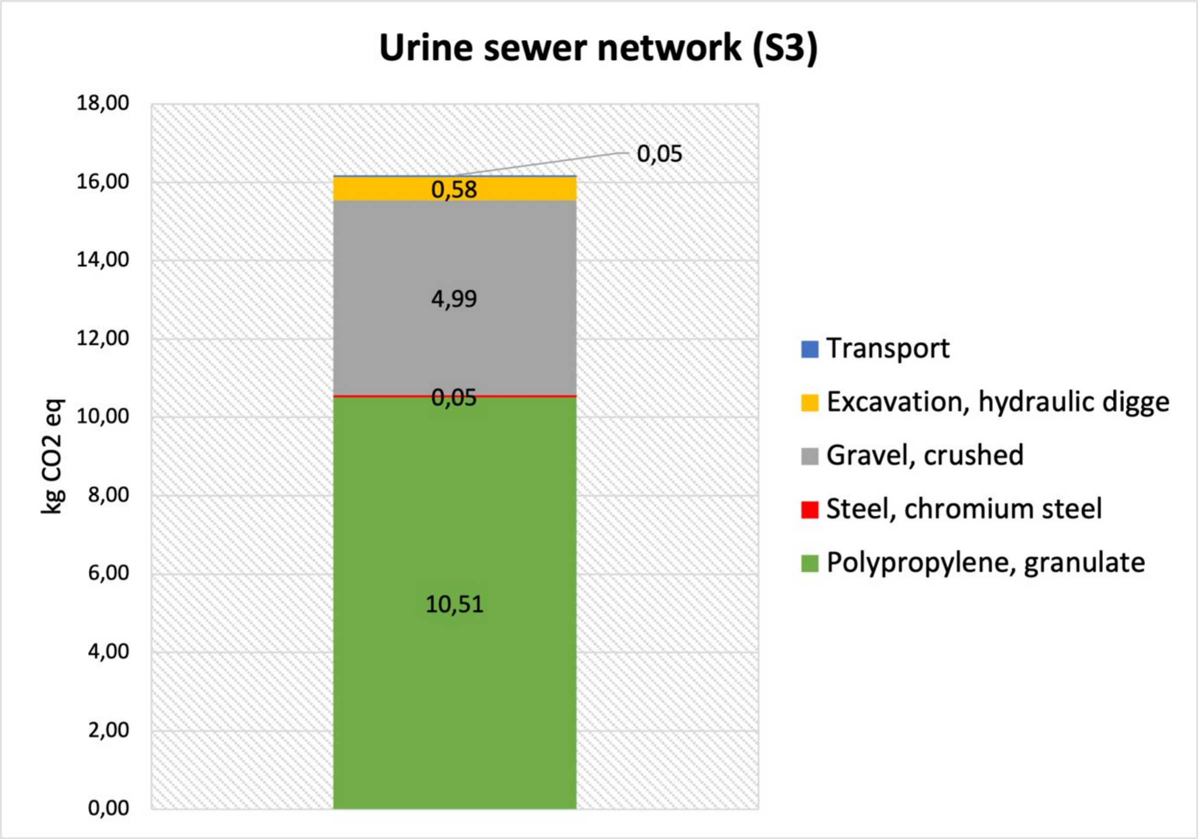

Figure S14: Urine sewer network for scenario 3

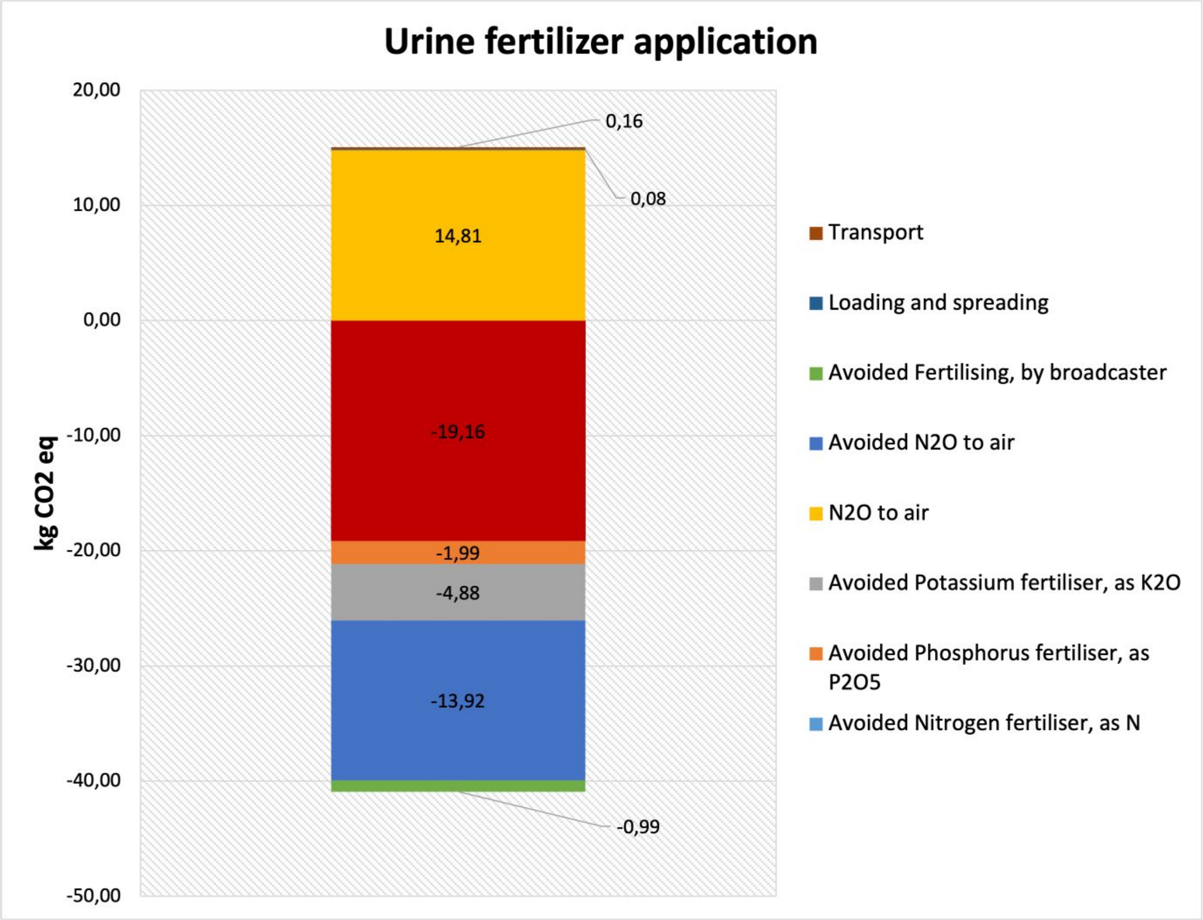

Figure S15: Breakdown of the urine fertilizer unit process for each scenario, showing burdens from inputs (materials, N_2O emissions, loading and spreading, transport) and credits from synthetic fertilizer substitution (N, P_2O_5, K_2O) and avoided N_2O emissions.

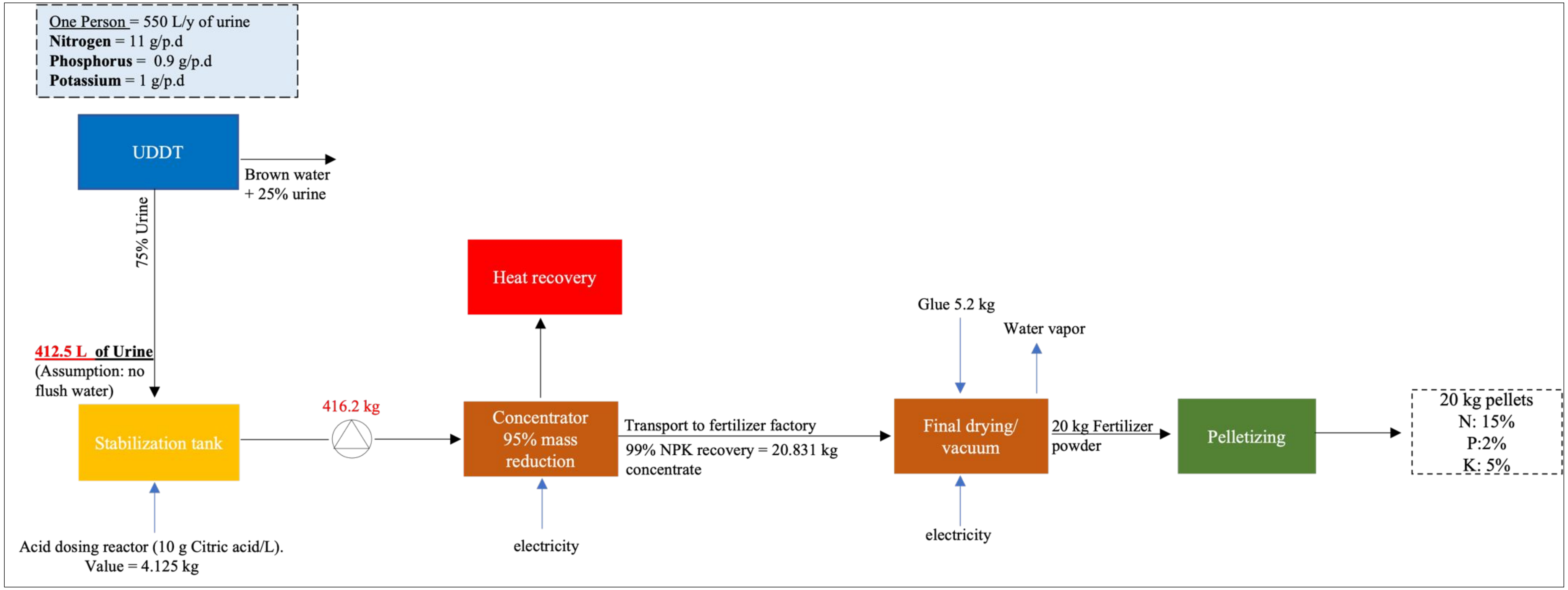

Figure S16: Schematic diagram of the primary unit process of the urine recycling system scenario 1. Energy recovery is achieved through heat recovery using a heat exchanger, which differs between the three scenarios. Each unit process is represented by a distinct
